# Macrohardness-Based Spatial Homogeneity Analysis of TIG-Welded AISI 304 Stainless Steel

**DOI:** 10.3390/ma19214708

**Published:** 2026-11-05

**Authors:** Saulius Baskutis, Yaroslav Kusyi, Lina Kavaliauskiene, Jolanta Baskutiene, Tomas Kačinskas, Oleh Onysko

**Affiliations:** 1 Department of Production Engineering, Faculty of Mechanical Engineering and Design, Kaunas University of Technology, Studentu St. 56, 51424 Kaunas, Lithuania; saulius.baskutis@ktu.lt (S.B.); lina.kavaliauskiene@ktu.lt (L.K.); jolanta.baskutiene@ktu.lt (J.B.); 2 Department of Robotics and Integrated Mechanical Engineering Technologies, Lviv Polytechnic National University, Bandera St. 12, 79013 Lviv, Ukraine; yaroslav.m.kusyi@lpnu.ua; 3 Department of Computerized Mechanical Engineering, Ivano-Frankivsk National Technical University of Oil and Gas, Karpatska St. 15, 76000 Ivano-Frankivsk, Ukraine; onysko.oleg@gmail.com

**Keywords:** TIG welding, AISI 304 stainless steel, macrohardness, material homogeneity criterion, coefficient of variation, Weibull homogeneity coefficient

## Abstract

**Highlights:**

**The main findings**

- Specimens 1 and 3 exhibited contrasting tensile responses despite comparatively similar mean HRBW ranges.
- Specimen 1, which fractured through the weld region, exhibited greater local macrohardness dispersion than Specimen 3.
- CV and WHC differentiated the two welded joints more clearly than mean HRBW alone.
- The largest between-specimen differences in macrohardness dispersion occurred within the heat-affected zone.

**Implications of the main findings**

- Statistical macrohardness mapping provides discriminatory information beyond conventional mean-hardness profiles.
- CV and WHC enable direct spatial comparison of welded joints exhibiting different mechanical responses.
- The contrasting-case comparison supports the use of hardness-dispersion descriptors for complementary welded-joint characterization.
- The approach remains complementary to tensile testing and metallography and is not presented as a stand-alone predictor of weld performance.

**Abstract:**

This study evaluates a material homogeneity criterion (MHC)-based workflow for the spatial characterization of macrohardness variability in tungsten inert gas (TIG) welded AISI 304 stainless-steel joints produced using 308LSi filler metal. Five welded specimens produced under different welding conditions were first evaluated by transverse tensile testing. Two specimens representing contrasting mechanical responses were subsequently selected for identical spatial macrohardness mapping: Specimen 1 exhibited the lowest ultimate tensile strength (610 MPa), 24% elongation, and fracture through the weld region, whereas Specimen 3 exhibited the highest ultimate tensile strength (684 MPa), 41% elongation, and fracture in the base material. For each specimen, 1000 Rockwell macrohardness (HRBW) measurements were obtained along twenty transverse measurement lines spanning the base material (BM), heat-affected zone (HAZ), and fusion zone (FZ). Mean hardness ranges were comparatively similar for Specimens 1 and 3 (92.04–95.70 and 92.00–95.50 HRBW, respectively). Meanwhile, the coefficient of variation (CV) and Weibull homogeneity coefficient (WHC) provided greater discrimination between the two joints. Specimen 1 exhibited CV values of 0.534–1.433% and WHC values of 81.20–216.95, whereas Specimen 3 exhibited CV values of 0.542–1.076% and WHC values of 107.87–214.12. The largest between-specimen difference was observed within the HAZ, where Specimen 1 exhibited substantially greater macrohardness dispersion than Specimen 3. These results show that statistical treatment of repeated macrohardness measurements can reveal spatial differences between welded joints that are less evident from mean-hardness profiles alone. The comparison does not establish a general predictive relationship between hardness dispersion and tensile performance but provides experimental evidence supporting the discriminatory value of spatial CV and WHC mapping for the two investigated contrasting cases. Optical metallography provides complementary qualitative microstructural characterization, while quantitative relationships between microstructural parameters and macrohardness dispersion remain outside the scope of the present study.

## 1. Introduction

Ensuring sufficiently uniform material response across welded joints is important because the fusion zone (FZ), heat-affected zone (HAZ), and base material (BM) may exhibit different mechanical and microstructural characteristics [1,2]. Similar considerations apply to coatings [3,4,5] and mechanically processed materials [6,7,8,9]. In welded structures, spatial variations in microstructure and mechanical response may influence local strength, ductility, residual-stress development, and susceptibility to crack initiation [10,11]. In stainless-steel weldments, such variations are particularly relevant because local chemical and microstructural changes may also affect corrosion behaviour [12,13,14].

**Figure 1:**
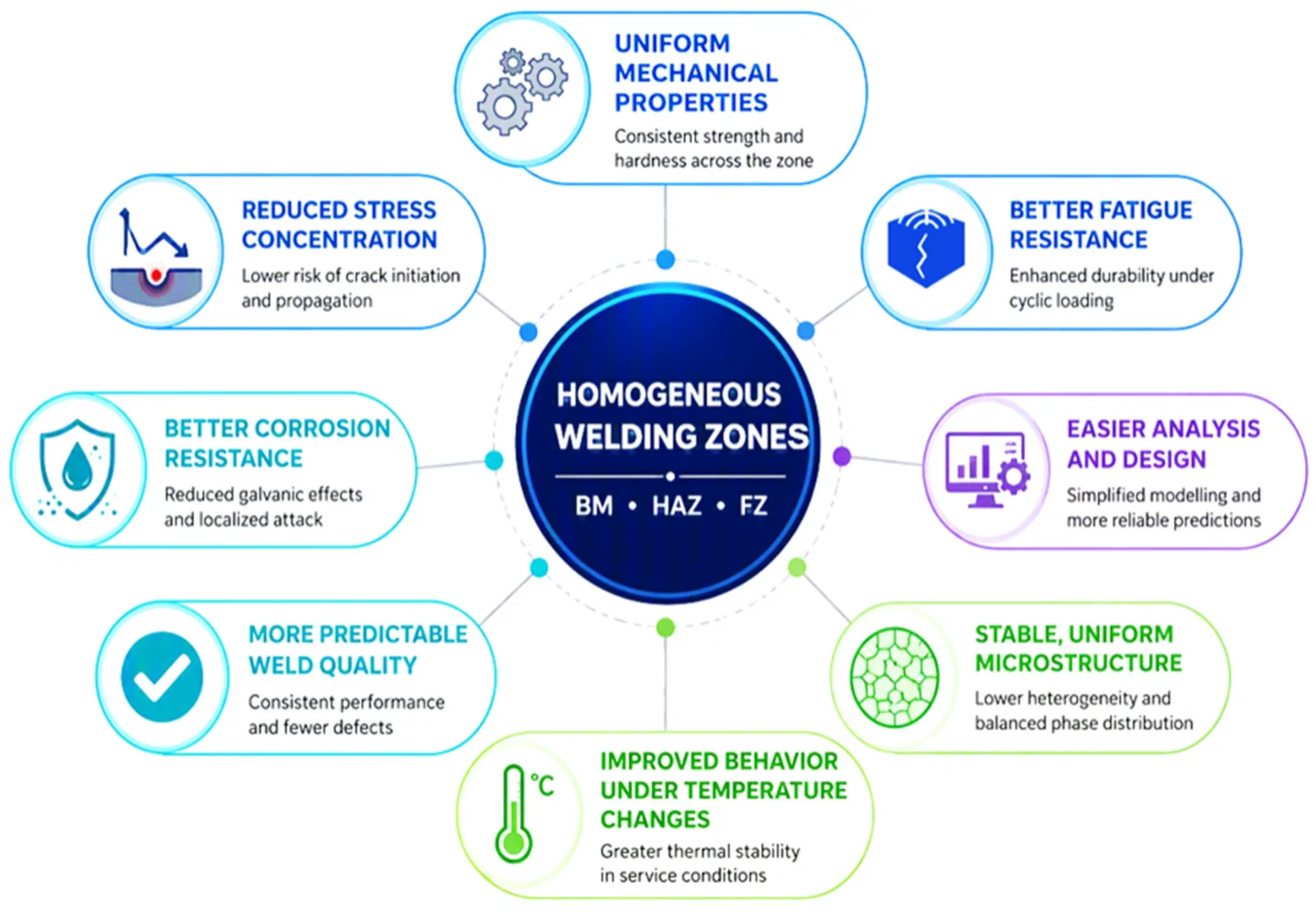
Key advantages of homogeneous welding zones (BM, HAZ and FZ).

The HAZ is of particular interest because it experiences substantial thermal exposure without melting. Depending on the thermal cycle, austenitic stainless steels may exhibit grain growth and other local microstructural changes in this region [15,16,17,18]. Grain coarsening close to the fusion boundary and, under suitable thermal conditions, grain-boundary carbide precipitation have been reported for welded stainless steels [19,20,21]. Sensitization of austenitic stainless steels may occur after exposure to critical temperature ranges and can increase susceptibility to intergranular corrosion [22]. Consequently, control of welding heat input and thermal conditions is important for limiting undesirable changes in the HAZ [23,24]. Figure 1 summarizes the general relevance of spatially uniform material response across the principal weld regions.

Austenitic stainless-steel welded joints are widely used in applications requiring corrosion resistance, weldability, and stable mechanical performance [25,26,27,28,29]. AISI 304 is among the most widely applied austenitic stainless steels because of its combination of mechanical properties, weldability, and corrosion resistance [1,30]. Since welding produces a spatially non-uniform thermal history, characterization methods capable of resolving local variations across the welded joint are important for understanding the resulting material response.

A range of conventional methods is available for welded-joint characterization, including ultrasonic, radiographic, eddy-current, magnetic-particle, metallographic, and mechanical testing [31,32,33,34,35]. These methods provide different types of information and should be regarded as complementary. Hardness testing offers a comparatively simple means of obtaining spatially resolved information on the local indentation response and is widely used for characterizing welded materials [17,36,37,38]. However, conventional evaluation based primarily on individual or mean hardness values may not fully describe the local statistical dispersion of repeated measurements.

Statistical analysis of repeated hardness measurements has therefore been investigated as an additional material-characterization approach [6,39,40,41]. Within this framework, the Lebedev-Muzyka hardness (LM-hardness) methodology evaluates not only the absolute hardness level but also the statistical distribution of repeated macrohardness measurements [39,40,41]. Previous studies have applied parameters such as the coefficient of variation and Weibull-based homogeneity descriptors to characterize the dispersion of mechanical-response measurements under different material and manufacturing conditions [6,39,42].

The LM-hardness method has previously been described as quasi-non-destructive because the material alteration is limited to localized indentation marks [42]. However, macrohardness measurements produce permanent surface impressions and therefore do not meet a strict definition of conventional non-destructive testing. The practical invasiveness of the procedure depends on indentation size and number, surface preparation, component geometry, material properties, and subsequent service conditions. Accordingly, the present study treats the method as a localized, minimally destructive indentation-based characterization approach rather than as conventional non-destructive testing (NDT).

A fundamental feature of the LM-hardness methodology is that it can be implemented using conventional macrohardness techniques, including Brinell, Rockwell, and Vickers testing [43]. Macrohardness measurements sample a substantially larger material volume than micro- or nanoindentation and therefore represent an integrated local mechanical response involving multiple grains and microstructural constituents. Statistical evaluation of repeated macrohardness measurements can consequently provide information on the spatial variability of this indentation response that is not available from the mean value alone [43,44].

Applications of LM-hardness-based statistical analysis have previously been reported for carbon and low-alloy steels, heat-resistant materials, and components subjected to manufacturing operations such as machining [39,41,42]. However, its spatial implementation across the FZ–HAZ–BM sequence of TIG-welded AISI 304 stainless steel remains insufficiently investigated. In particular, it remains unclear whether line-by-line statistical evaluation of repeated macrohardness measurements can reveal spatial variations in hardness dispersion that are not evident from conventional mean-hardness profiles.

In the present study, the LM-hardness concept, the Weibull homogeneity coefficient WHC(*m*), and the coefficient of variation CV(*ν*) are treated as established statistical tools [6,39,40,42]. The methodological contribution lies in their spatial implementation across predefined transverse measurement lines extending through the BM–HAZ–FZ–HAZ–BM sequence of TIG-welded AISI 304 joints. Repeated Rockwell macrohardness measurements are used to characterize both the local hardness level and the statistical dispersion of hardness at each spatial position.

The purpose of the study is not to interpret hardness dispersion as a direct quantitative measure of physical microstructural heterogeneity, degradation, damage, structural integrity, or remaining service life. Instead, CV(*ν*) and WHC(*m*) are used as statistical descriptors of macrohardness variability. Optical metallography provides complementary qualitative microstructural context, while transverse tensile testing provides specimen-level mechanical characterization. No assumption is made that the hardness-dispersion descriptors constitute direct predictors of tensile performance or specific microstructural mechanisms.

Accordingly, the aim of this study is to determine whether spatial statistical analysis of repeated Rockwell macrohardness measurements provides discriminatory information beyond conventional mean-hardness profiles when applied to TIG-welded AISI 304 joints exhibiting contrasting mechanical responses. Five welded specimens produced under different TIG conditions were initially characterized by transverse tensile testing. Specimen 1, which exhibited the lowest tensile strength and elongation and fractured through the weld region, and Specimen 3, which exhibited the highest tensile strength and elongation and fractured in the base material, were subsequently selected for identical spatial macrohardness mapping. Mean HRBW, CV(*ν*), and WHC(*m*) were evaluated at corresponding positions across the BM–HAZ–FZ–HAZ–BM sequence of both specimens. The working hypothesis was that dispersion-based descriptors of repeated macrohardness measurements could reveal spatial differences between the two contrasting welded joints that are less evident from mean hardness alone. The novelty of the work therefore lies not in the individual statistical indicators themselves, but in their integration with dense transverse Rockwell macrohardness mapping and in the direct position-by-position comparison of two welded joints exhibiting distinctly different mechanical responses.

## 2. Materials and Methods

### 2.1. Materials

The investigated material consisted of 10 mm-thick AISI 304 austenitic stainless-steel sheets with dimensions of 250 mm × 100 mm × 10 mm. Table 1 and Table 2 present the manufacturer-declared chemical composition and nominal mechanical properties of the AISI 304 base material (Outokumpu Stainless AB, Degerfors, Sweden) and OK Tigrod 308LSi filler rod, respectively (ESAB Welding & Cutting Products, Hanover, York County, PA, USA).

**Figure 2:**
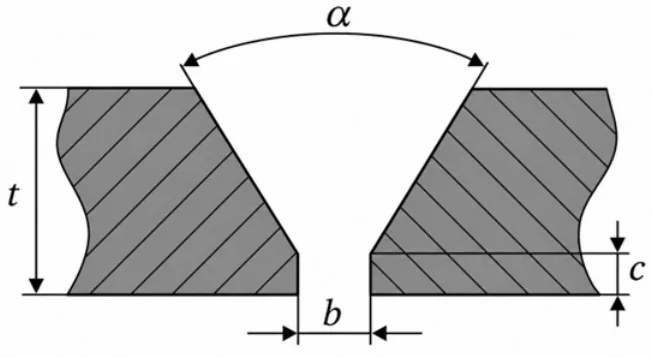
The geometry of sheet edge: α—an angle of the groove edge (60°); t—the thickness of the weldment (10 mm); b—root opening (3 mm); c—thickness of root face (2 mm).

Stainless steel sheets for welding were prepared to the required dimensions according to the requirements of the standard EN ISO 9692-1:2013 [45] for a single Y-butt weld with broad root face and backing run. The groove α with an angle of 60° was formed by a Doosan DNM 5700 CNC vertical milling machine (Doosan Machine Tools Co., Ltd., Changwon, Republic of Korea). The geometry of the edges of the welded sheets is presented in Figure 2. A gap (b) of 3 mm was left between the edges of the sheets during welding. Before welding, the specimens were polished and degreased with ethanol (Merck KGaA, Darmstadt, Germany) to remove any surface dirt, oxides or dust.

### 2.2. Methods

#### 2.2.1. Welding

The AISI 304 stainless-steel joints were produced using the gas tungsten arc welding (GTAW) process, also known as tungsten inert gas (TIG; process 141) welding, with argon shielding gas (AGA, Linde Gas AB, Solna, Sweden) using a MasterTig 335 ACDC welding machine (KEMPPI OY, Lahti, Finland). A 2.4 mm diameter WL20 tungsten electrode (KEMPPI OY, Lahti, Finland) and 2.0 mm diameter OK Tigrod 308LSi low-carbon, high-silicon austenitic stainless-steel filler rods (ESAB Welding & Cutting Products, Hanover, York County, PA, USA) were used. Pure argon (99.9%) was supplied as the shielding gas at a flow rate of 20 L/min. Direct current was used during welding.

**Figure 3:**
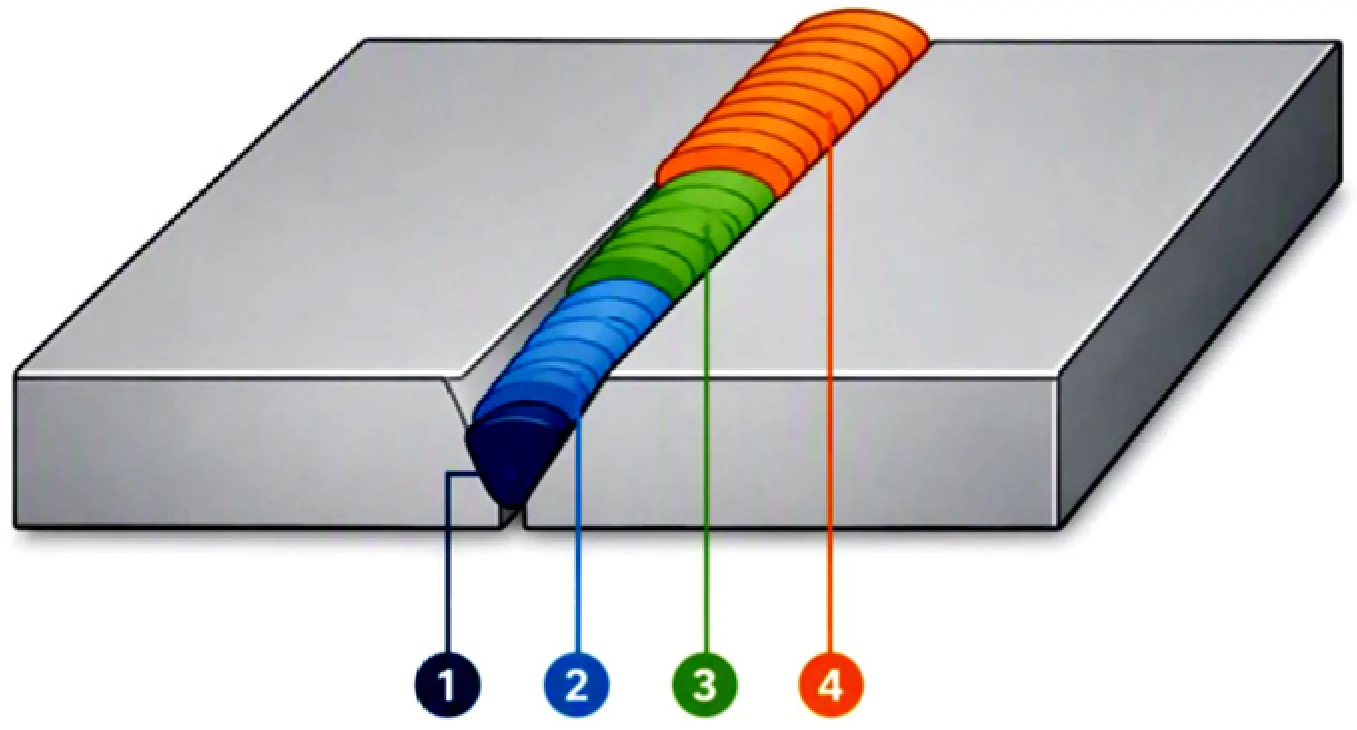
Four-pass welding sequence used for the experimental joints: 1—root pass; 2—hot pass; 3—fill pass; 4—cap pass.

Each welded joint was deposited in four consecutive passes: root, hot, fill, and cap, as illustrated in Figure 3. The hot, fill, and cap passes were performed using the same parameter set within each individual specimen.

The general procedural requirements for joint preparation, welding sequence, welding position, and interpass-temperature control were based on the applicable TIG welding procedure specification. The procedure specified a nominal groove angle of approximately 60° and a maximum interpass temperature of 150 °C. The actual electrical and travel parameters used for the five experimental specimens are reported in Table 3.

The principal welding parameters are summarized in Table 3. Welding current, voltage, and travel speed were controlled according to the selected welding condition. The calculated heat input, (*Q*), was determined using a TIG thermal efficiency factor of *η* = 0.6:

(1) $$Q=\frac{\mathrm{\eta UI}}{v}$$

where (*U*) is the welding voltage (V), (*I*) is the welding current (A), and (*v*) is the travel speed (mm/s). The calculated heat input is expressed in J/mm. The arc length was maintained at approximately 3 mm throughout welding.

Based on the transverse tensile-test results described in Section 3.1, Specimens 1 and 3 were selected for detailed spatial macrohardness characterization because they represented the most contrasting mechanical responses among the five investigated welding conditions. Specimen 1 exhibited the lowest ultimate tensile strength and elongation and fractured through the weld region, whereas Specimen 3 exhibited the highest ultimate tensile strength and elongation and fractured in the base material. The purpose of selecting these two specimens was therefore to determine whether identical spatial macrohardness mapping and statistical analysis could reveal differences between welded joints exhibiting distinctly different tensile responses.

Specimen 1 was produced using 11 V, 90 A, and a travel speed of 3.5 mm/s for the root pass, corresponding to a calculated heat input of 169.71 J/mm. The hot, fill, and cap passes were performed at 11 V, 120 A, and 5.0 mm/s, corresponding to a heat input of 158.40 J/mm. Specimen 3 was produced using 14 V, 110 A, and 3.5 mm/s for the root pass, corresponding to 264.00 J/mm, whereas the hot, fill, and cap passes were performed at 14 V, 130 A, and 5.0 mm/s, corresponding to 218.40 J/mm.

The complete welding thermal cycles and cooling rates were not directly monitored using thermocouples or another temperature-acquisition system. Accordingly, no quantitative cooling-rate values are reported, and the present comparison does not establish a causal relationship between individual welding parameters, thermal history, and the subsequently determined macrohardness-dispersion parameters.

**Figure 4:**
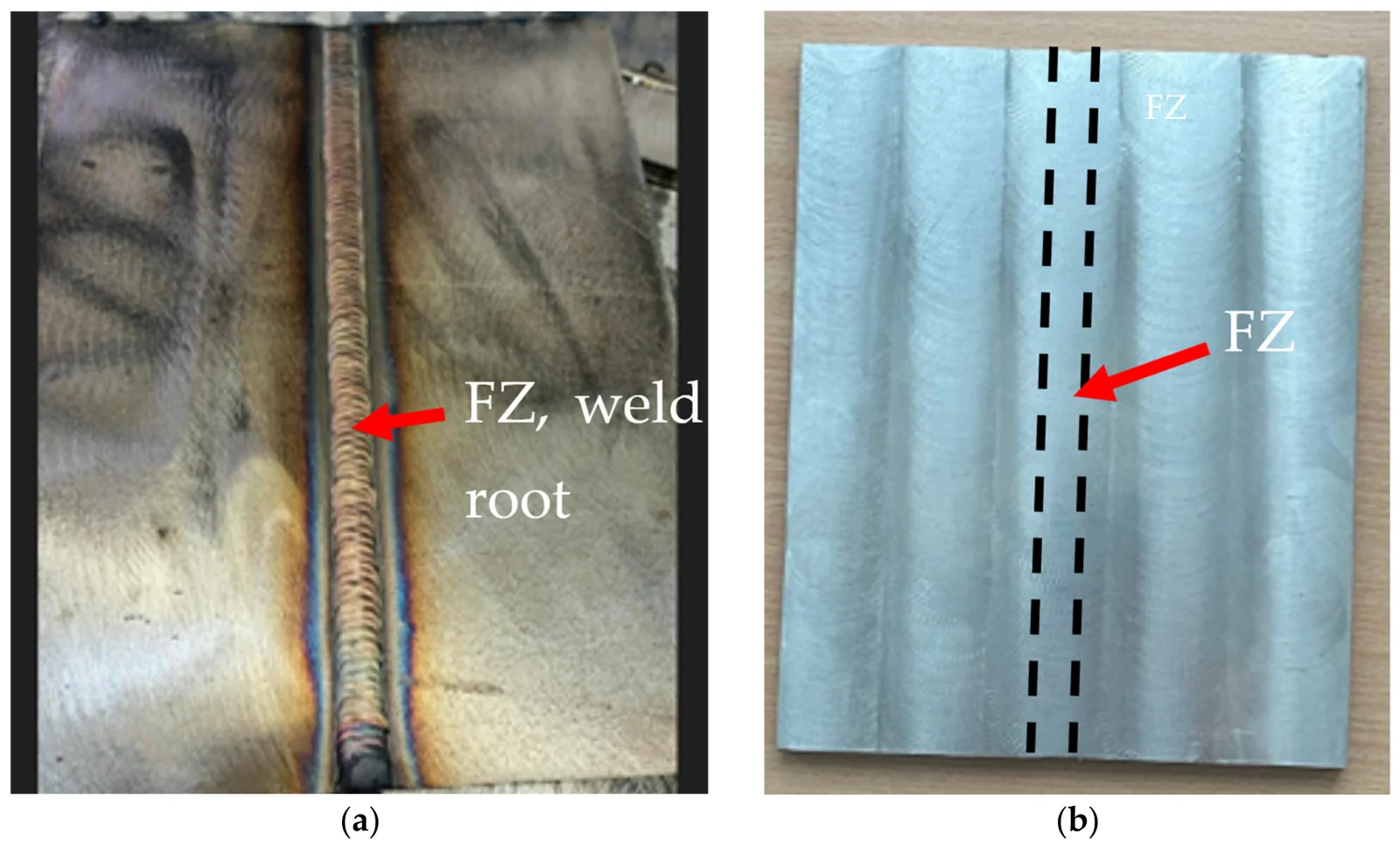
Specimen 3 used for macrohardness testing: (**a**) after welding; (**b**) after precision milling and surface preparation. The same preparation procedure was applied to Specimen 1 before spatial macrohardness mapping.

Following welding, Specimens 1 and 3 were prepared for spatial Rockwell macrohardness measurements using the same surface-preparation procedure. The weld reinforcement was removed by precision milling to obtain a flat measurement surface (Figure 4b), after which the prepared surfaces were cleaned to remove oxide scale, machining residues, foreign matter, and lubricants.

Precision milling was used as a laboratory specimen-preparation step to provide a sufficiently flat and reproducible surface for Rockwell macrohardness testing [46]. This preparation step is not an intrinsic component of the statistical analysis itself; however, application of the macrohardness-based procedure requires surface conditions suitable for reliable indentation measurements. Depending on the geometry and surface condition of an investigated component, local surface preparation may therefore be necessary.

For macrohardness testing, the specimen was placed on a rigid support with the test surface positioned normal to the indenter axis and the direction of the applied force. The specimen was firmly supported to prevent displacement during indentation. All macrohardness measurements were performed at an ambient temperature of 20 ± 2 °C and a relative humidity of 50 ± 5%.

#### 2.2.2. Macrohardness Analysis

Hardness testing provides a practical means of characterizing the resistance of a material to localized plastic deformation under indentation loading [43]. The measured hardness value depends on the material response as well as on the selected test method, indenter geometry, applied load, and measurement scale and therefore should be regarded as an engineering response parameter rather than a fundamental material constant [43,47]. Relationships between hardness and other mechanical properties, including yield strength and ultimate tensile strength, are material- and condition-dependent and should not be assumed to be universal [43].

Hardness measurements may be performed at macro-, micro-, and nanoscale, with each scale sampling different material volumes and structural features [43]. Micro- and nanoindentation can be sensitive to individual grains, grain boundaries, phases, or other local constituents, whereas macrohardness measurements sample a substantially larger material volume and therefore provide a more spatially integrated indentation response [43,44,48]. In the present study, Rockwell macrohardness was selected because the objective was to evaluate the statistical dispersion of repeated hardness measurements across predefined regions of the welded specimen rather than to characterize individual microstructural constituents.

Beyond the mean hardness value, repeated measurements can be evaluated statistically to describe the local dispersion of the indentation response. In the present study, two established statistical descriptors were used: the Weibull homogeneity coefficient, WHC(*m*), and the coefficient of variation, CV(*ν*) [6,39,40]. These parameters are interpreted strictly as descriptors of macrohardness dispersion and are not treated as direct quantitative measures of physical microstructural heterogeneity, degradation, damage, or structural integrity.

The WHC(*m*) parameter was calculated from the logarithmic dispersion of the measured hardness values according to [6]:

(2) $$m=\frac{d\left(n\right)}{2.30259\cdot S\left({l\mathrm{og}}_{10}H\right)}$$

where

(3) $$S\left(lg\left(H\right)\right)=\sqrt{\frac{1}{n-1}\cdot \sum_{i=1}^{n}{\left({l\mathrm{og}}_{10}\left(H_{i}\right)-\bar{{l\mathrm{og}}_{10}\left(H\right)}\right)}^{2}}$$

and:

(4) $$\bar{{l\mathrm{og}}_{10}\left(H\right)}=\frac{1}{n}\cdot \sum_{i=1}^{n}{l\mathrm{og}}_{10}\left(H_{i}\right)$$

Here, *H_i_* is the measured macrohardness value at the *i_-__th_* indentation point, *n* is the number of observations included in the calculation, and *d*(*n*) is the measurement-number-dependent coefficient used in the adopted WHC formulation [6]. For the present analysis, *n* = 50 for each measurement line.

Within this formulation, lower WHC(*m*) values correspond to greater dispersion of the logarithmically transformed hardness measurements, whereas higher values indicate a comparatively narrower local hardness distribution. Previous LM-hardness studies have used *m* = 10 as a practical boundary between heterogeneous and homogeneous material states [6,49]. However, this threshold has not been independently validated for TIG-welded AISI 304/308LSi joints and is therefore not used as an absolute classification criterion in the present study. WHC(*m*) is instead interpreted as a continuous comparative descriptor across the investigated FZ–HAZ–BM sequence.

The coefficient of variation, CV(*ν*), was calculated as:

(5) $$\nu =\frac{\sqrt{\frac{1}{n-1}\cdot \sum_{i=1}^{n}{\left(H_{i}-\bar{H}\right)}^{2}}}{\bar{H}}\cdot 100\;[\%]$$

where $\bar{H}$ is the arithmetic mean macrohardness of the corresponding measurement line. CV(*ν*) expresses the standard deviation relative to the mean hardness and therefore provides a direct measure of relative hardness dispersion.

WHC(*m*) and CV(*ν*) were calculated from the same local hardness datasets and should therefore not be regarded as statistically independent validation parameters. Rather, they provide two different numerical representations of the dispersion of the same underlying HRBW measurements. CV(*ν*) describes relative scatter on the original hardness scale, whereas WHC(*m*) characterizes dispersion in the logarithmically transformed hardness data.

For each predefined transverse measurement line, 50 HRBW measurements were processed independently. The resulting mean HRBW, CV(*ν*), and WHC(*m*) values were assigned to the corresponding spatial position within the FZ–HAZ–BM sequence. This procedure was applied independently to both selected welded specimens. Twenty measurement lines were evaluated in each specimen, resulting in 1000 individual HRBW measurements per specimen and 2000 measurements in the comparative dataset. The measurements represent within-specimen spatial subsampling and should not be interpreted as independent welded-joint replicates.

**Figure 5:**
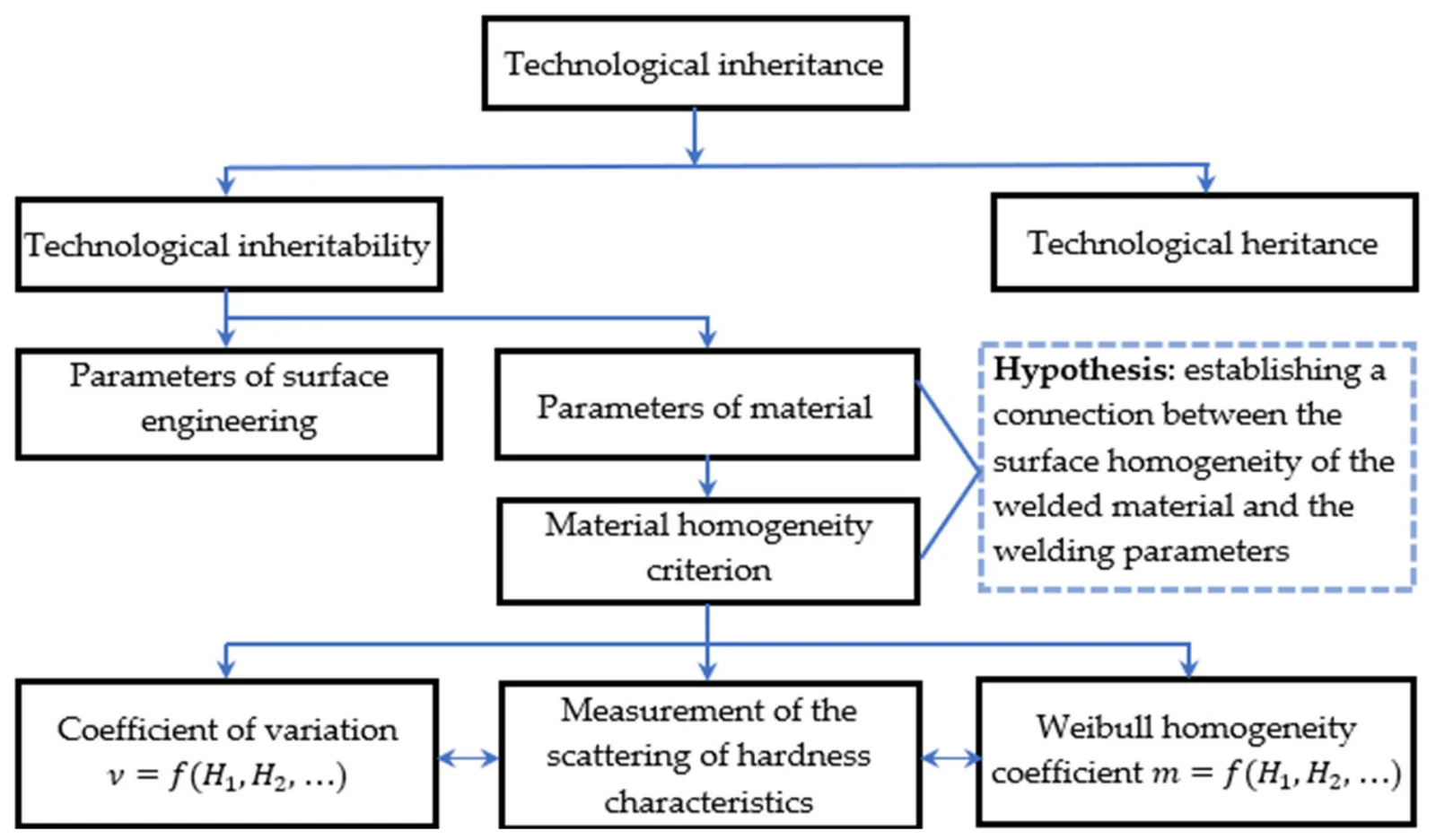
Conceptual framework for the statistical assessment of material homogeneity based on the spatial dispersion of hardness characteristics. The framework relates technological and material factors to hardness variability and its statistical characterization using the coefficient of variation, CV(*ν*), and the Weibull homogeneity coefficient, WHC(*m*).

The methodological contribution of the present work lies in the weld-specific spatial implementation of these established hardness-based statistical descriptors rather than in the introduction of a new statistical parameter. The framework used in this study is illustrated schematically in Figure 5.

The macrohardness-based procedure involves permanent indentation marks and, in the present laboratory implementation, surface preparation by precision milling. It is therefore treated as a localized, minimally destructive indentation-based characterization method rather than as conventional non-destructive testing. Its applicability to engineering components depends on surface condition, accessibility, component geometry, material properties, loading conditions, and the acceptability of permanent local indentations [46].

#### 2.2.3. Statistical Analysis and Uncertainty Assessment

For each of the two selected welded specimens (Specimens 1 and 3), twenty measurement lines were defined symmetrically across the investigated welded specimen and designated −10 to −1 on the left-hand side and +1 to +10 on the right-hand side of the weld centerline. Their respective positions ranged from −20 to −2 mm and from +2 to +20 mm, with a spacing of Δ*x* = 2.0 mm between adjacent lines. Fifty individual HRBW measurements were obtained along each measurement line.

Thus, 1000 hardness observations were obtained for each specimen, and 2000 HRBW measurements were included in the comparative spatial analysis.

Each measurement line represents a predefined spatial position within a given welded specimen, whereas the 50 measurements obtained along that line constitute local within-line subsampling. The two selected specimens represent two contrasting welding conditions and mechanical responses; they should not be interpreted as replicate welds produced under identical conditions.

For each measurement line of each specimen (*n* = 50), the arithmetic mean, sample standard deviation (*SD*), standard error of the mean (*SE*), and two-sided 95% confidence interval (*CI*) of the mean were calculated as follows:

(6) $$\bar{H}=\frac{1}{n}\sum_{i=1}^{n}H_{i}$$

(7) $$s=\sqrt{\frac{1}{n-1}\sum_{i=1}^{n}{\left(H_{i}-\bar{H}\right)}^{2}}$$

(8) $$\mathrm{SE}=\frac{s}{\sqrt{n}}$$

(9) $${\mathrm{CI}}_{95\%}=\bar{H}\pm t_{0.975,n-1}\mathrm{SE}$$

where *H_i_* is the individual HRBW measurement, $\bar{H}$ is the arithmetic mean, *s* is the sample standard deviation, and *n* = 50 is the number of observations within the corresponding measurement line. The 95% *CI* of the mean was calculated using the Student-*t* distribution with *n*−1 = 49 degrees of freedom.

CV(*ν*) and WHC(*m*) were calculated according to Equations (2)–(5) using the adopted LM-hardness formulation [6,39,40]. Because the sampling distributions of these dispersion-based estimators cannot be assumed to be normally distributed, their uncertainty was evaluated by non-parametric bootstrap resampling [50]. For each measurement line of each specimen, the corresponding set of 50 experimentally measured HRBW values was resampled with replacement 20,000 times. CV(*ν*) and WHC(*m*) were recalculated for each bootstrap sample, and the 2.5th and 97.5th percentiles of the resulting bootstrap distributions were taken as the limits of the corresponding 95% percentile-bootstrap confidence intervals.

The sensitivity of CV(*ν*) and WHC(*m*) to the number of observations included in the calculation was additionally evaluated separately for each specimen by repeated random subsampling without replacement. Subsets of *n* = 15, 20, 25, 30, and 40 observations were drawn from the complete set of 50 measurements for each measurement line. For each sample size, specimen, and measurement line, 10,000 random subsets were generated, and CV(*ν*) and WHC(*m*) were recalculated for each subset. The resulting estimates were compared with those obtained from the complete *n* = 50 dataset.

The measurement-number-dependent coefficient *d*(*n*) corresponding to each sample size was applied in the WHC(*m*) calculation [6,39,40,51]:

*d*(15) = 1.0206,

*d*(20) = 1.0628,

*d*(25) = 1.0915,

*d*(30) = 1.1124,

*d*(40) = 1.1413,

*d*(50) = 1.1607.

The measurement lines and individual indentations represent spatial observations within two individual welded specimens produced under different welding conditions. They therefore provide a direct descriptive comparison between two contrasting welded joints but do not constitute replicated experiments within either welding condition. Accordingly, the reported confidence intervals quantify uncertainty associated with the local within-specimen hardness measurements and the corresponding statistical estimators; they do not quantify between-weld reproducibility.

The comparison between Specimens 1 and 3 was therefore interpreted descriptively using the spatial profiles of mean HRBW, CV(*ν*), and WHC(*m*), together with their associated confidence intervals. No population-level hypothesis tests were used to claim reproducible effects of welding condition, weld region, or mechanical performance because only one specimen from each selected welding condition was subjected to detailed macrohardness mapping. Independent replicate welds produced under identical conditions would be required for confirmatory inferential testing and for quantitative assessment of between-specimen reproducibility.

Statistical uncertainty associated with the sampled hardness distributions was considered separately from the metrological uncertainty of the Rockwell hardness-testing system. Rockwell measurements were performed in accordance with EN ISO 6508-1:2023 [46]. Instrument-specific measurement uncertainty cannot be reconstructed solely from the observed dispersion of repeated hardness measurements and requires the corresponding calibration, verification, resolution, indenter, and other relevant metrological information [46,47].

## 3. Results

### 3.1. Tensile Testing

**Figure 6:**
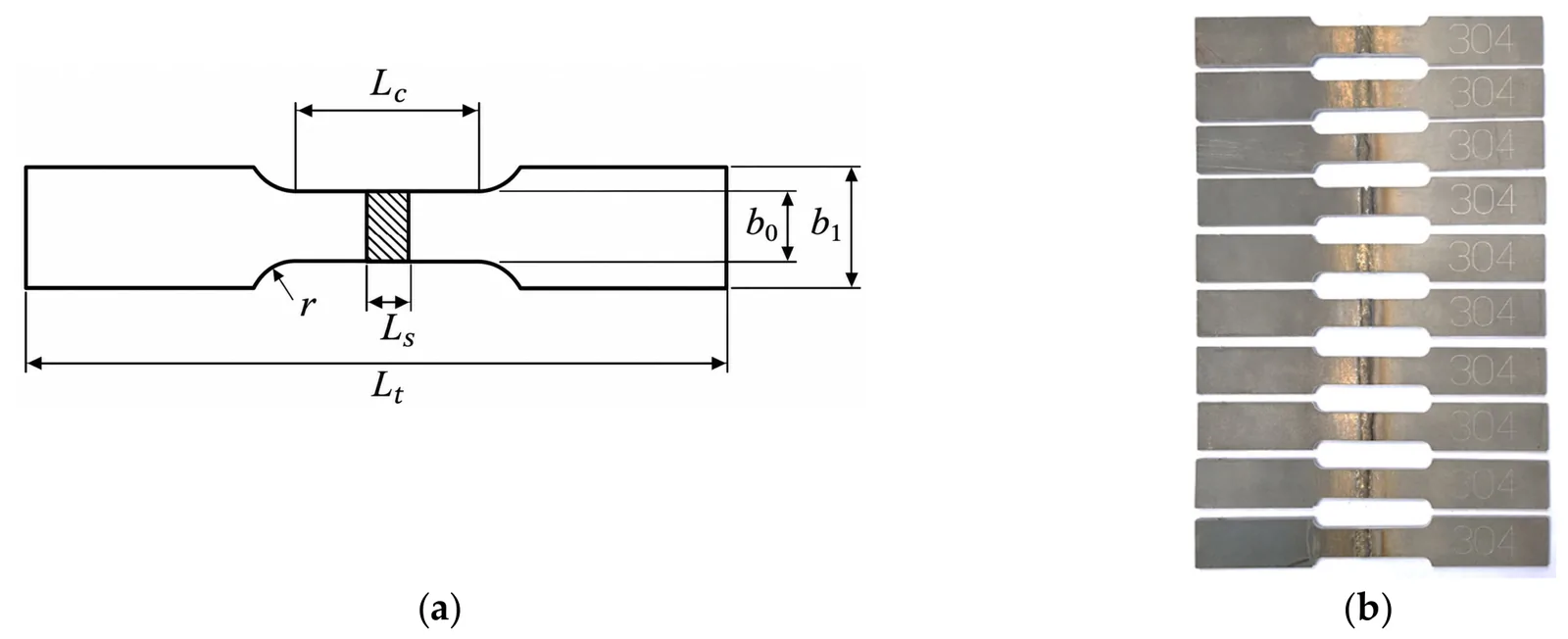
Tensile specimens: (**a**) specimen geometry: *L_t_*—total length (180 mm); *L_c_*—gauge length (60 mm); *L_s_*—maximum weld width (the diagonally hatched region in the center represents the weld metal (fusion zone); *b*_0_—reduced-section width (14 mm); *b*_1_—grip-section width (25 mm); *r*—shoulder radius (25 mm); (**b**) examples of prepared tensile specimens.

The welded joints were subjected to transverse tensile testing to characterize their mechanical response and fracture location. The tests were performed using a Tinius Olsen H100KT universal testing machine (Tinius Olsen Ltd., Redhill, UK). The tensile specimens, with a gauge length of 60 mm, were prepared and tested in accordance with ISO 4136:2012 [52], as shown in Figure 6.

**Figure 7:**
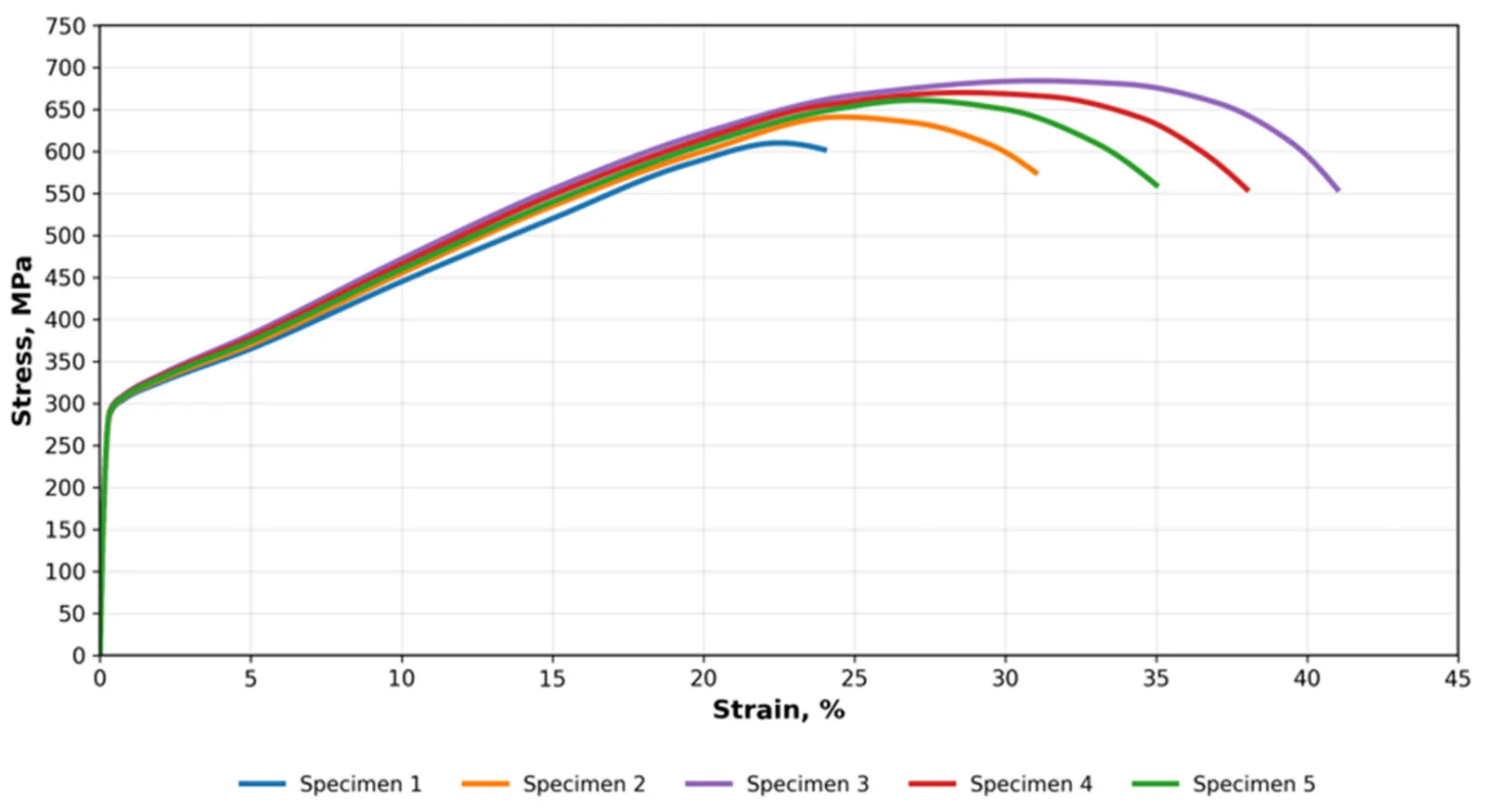
Stress–strain curves of the five investigated welded specimens.

The tensile stress–strain responses of the five welded AISI 304 specimens are presented in Figure 7.

The experimentally determined ultimate tensile strength (UTS), elongation, and fracture location of the five welded specimens are summarized in Table 4.

**Figure 8:**
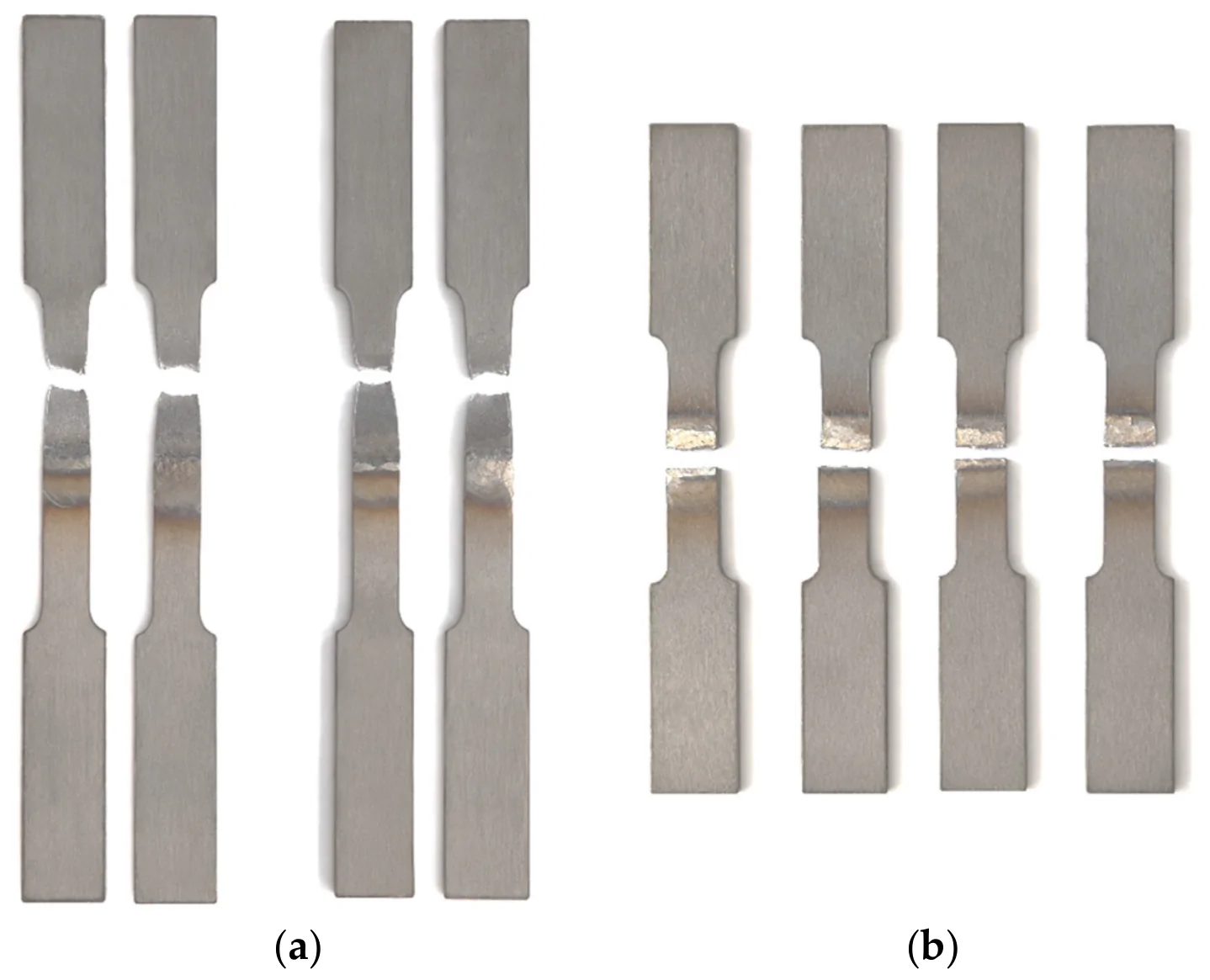
Representative fracture locations after tensile testing: (**a**) Specimen 3, fractured in the base material; (**b**) Specimen 1, fractured through the weld region.

Specimen 1 exhibited the lowest UTS (610 MPa) and elongation (24%) among the five investigated welding conditions and fractured through the weld region. Meanwhile, Specimens 2–5 fractured in the base material. Specimen 3 exhibited the highest UTS (684 MPa) and elongation (41%) of the investigated joints. Thus, Specimens 1 and 3 represented the two most contrasting tensile responses within the experimental set in terms of both tensile performance and fracture location. Representative fracture locations for Specimens 3 and 1 are shown in Figure 8.

The fracture of Specimen 1 through the weld region indicates that, under the corresponding welding condition, the welded region governed the final fracture location, whereas Specimens 2–5 fractured in the base material. Based on their contrasting tensile responses, Specimens 1 and 3 were selected for subsequent identical spatial macrohardness mapping and statistical analysis. Specimen 1 represents the condition with the lowest measured tensile strength and elongation and weld-region fracture, whereas Specimen 3 represents the condition with the highest measured tensile strength and elongation and base-material fracture.

The purpose of this selection was not to assume in advance that either specimen would exhibit greater or lower macrohardness dispersion. Rather, the two specimens were selected as contrasting mechanical-response cases to determine whether spatial analysis of mean HRBW, CV(*ν*), and WHC(*m*) could distinguish welded joints whose tensile behaviour differed substantially. Because only one welded specimen from each of these two welding conditions was subjected to detailed macrohardness mapping, the subsequent comparison should be interpreted as a between-condition case comparison rather than as a replicated statistical validation of the relationship between hardness dispersion and tensile performance.

### 3.2. Macrohardness Testing

Rockwell macrohardness measurements were performed across the BM–HAZ–FZ–HAZ–BM sequence of Specimens 1 and 3 in accordance with EN ISO 6508-1:2023 [46] using a VERZUS^®^ 750TM Rockwell hardness tester (INNOVATEST Europe BV, Maastricht, The Netherlands). A tungsten-carbide ball indenter with a diameter of 1.588 mm was applied under a total test force, *F*, of 980.7 N and a preliminary test force, *F*_0_, of 98.07 N.

For the HRBW scale, the Rockwell hardness value is determined from the permanent indentation depth according to [47]:

$$\mathrm{HRBW}=130-\;\frac{h}{0.002}$$

where *h* is the permanent indentation depth, in mm, measured after removal of the additional test force while maintaining the preliminary test force. The application time of the preliminary test force did not exceed 2 s.

Both selected welded specimens were investigated in detail by macrohardness mapping. The spatial HRBW measurements were performed according to the measurement scheme described in Section 2.2.2. Twenty transverse measurement lines were evaluated in each specimen, with 50 HRBW measurements obtained along each line. Thus, 1000 hardness observations were obtained for each specimen, resulting in a total of 2000 HRBW measurements used for comparative analysis.

**Figure 9:**
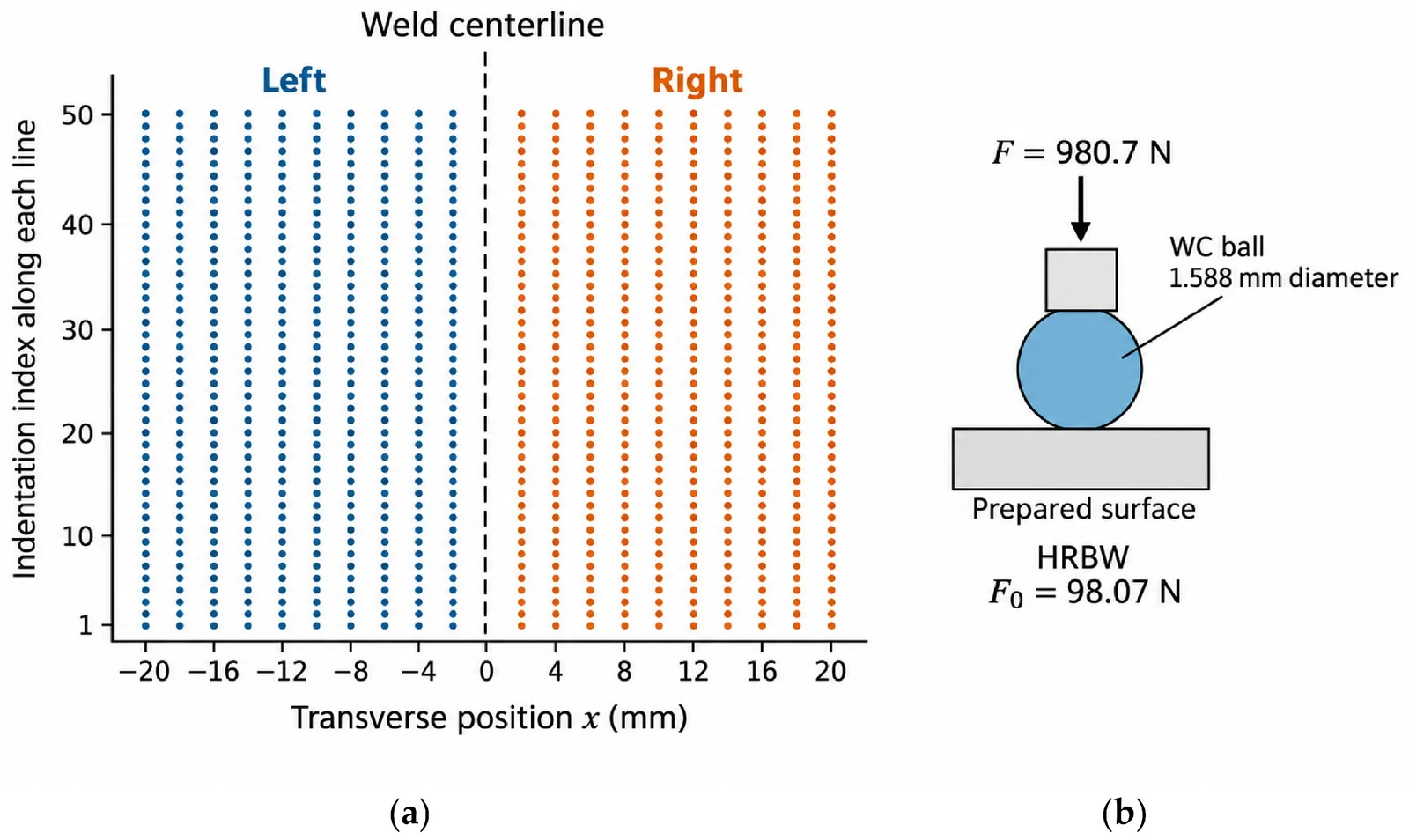
Common macrohardness measurement scheme applied to Specimens 1 and 3: (**a**) spatial arrangement of twenty transverse measurement lines, each containing 50 HRBW indentations, across the BM–HAZ–FZ–HAZ–BM sequence; (**b**) schematic representation of the HRBW indentation test using a 1.588 mm tungsten-carbide ball, a total test force of 980.7 N, and a preliminary test force of 98.07 N. The schematic is not drawn to scale.

The identical measurement arrangement enabled direct position-by-position comparison of the mean macrohardness and corresponding dispersion descriptors between Specimen 1, which exhibited the lowest tensile strength and weld-region fracture, and Specimen 3, which exhibited the highest tensile strength and base-material fracture. The distance between the centers of adjacent indentations was maintained in accordance with the requirements of EN ISO 6508-1:2023 [46]. The arrangement of the measurement lines across the FZ, HAZ, and BM is shown schematically in Figure 9.

The mean HRBW values obtained at each measurement position are presented in Table 5.

The mean macrohardness profiles of Specimens 1 and 3 were comparatively similar despite their distinctly different tensile responses. In Specimen 1, the mean HRBW values ranged from 92.04 to 95.70, whereas in Specimen 3 they ranged from 92.00 to 95.50 HRBW.

Both specimens exhibited the same general spatial tendency, with lower mean hardness values occurring primarily within the central FZ and higher values occurring within parts of the HAZ. The maximum mean value in Specimen 1 was 95.70 HRBW at −10 mm, while the maximum in Specimen 3 was 95.50 HRBW at the same position. The minimum mean value in Specimen 1 was 92.04 HRBW at +4 mm, whereas Specimen 3 exhibited a minimum of 92.00 HRBW at both −4 and +4 mm.

At most corresponding spatial positions, the difference in mean hardness between the two specimens was small. The largest local difference was observed at −6 mm within the FZ, where the mean hardness was 94.26 HRBW for Specimen 1 and 93.42 HRBW for Specimen 3, corresponding to a difference of 0.84 HRBW. At −8 mm within the HAZ, the corresponding values were 95.46 and 94.82 HRBW, giving a difference of 0.64 HRBW. The outer BM values of the two specimens were also closely comparable.

Consequently, conventional comparison based on the mean HRBW profiles alone provided only limited discrimination between Specimens 1 and 3 despite their substantial differences in tensile strength, elongation, and fracture location. This observation provides the basis for the subsequent comparison of CV(*ν*) and WHC(*m*), which evaluates whether the dispersion of repeated hardness measurements provides additional discriminatory information between the two welded joints.

### 3.3. Microstructure Analysis

**Figure 10:**
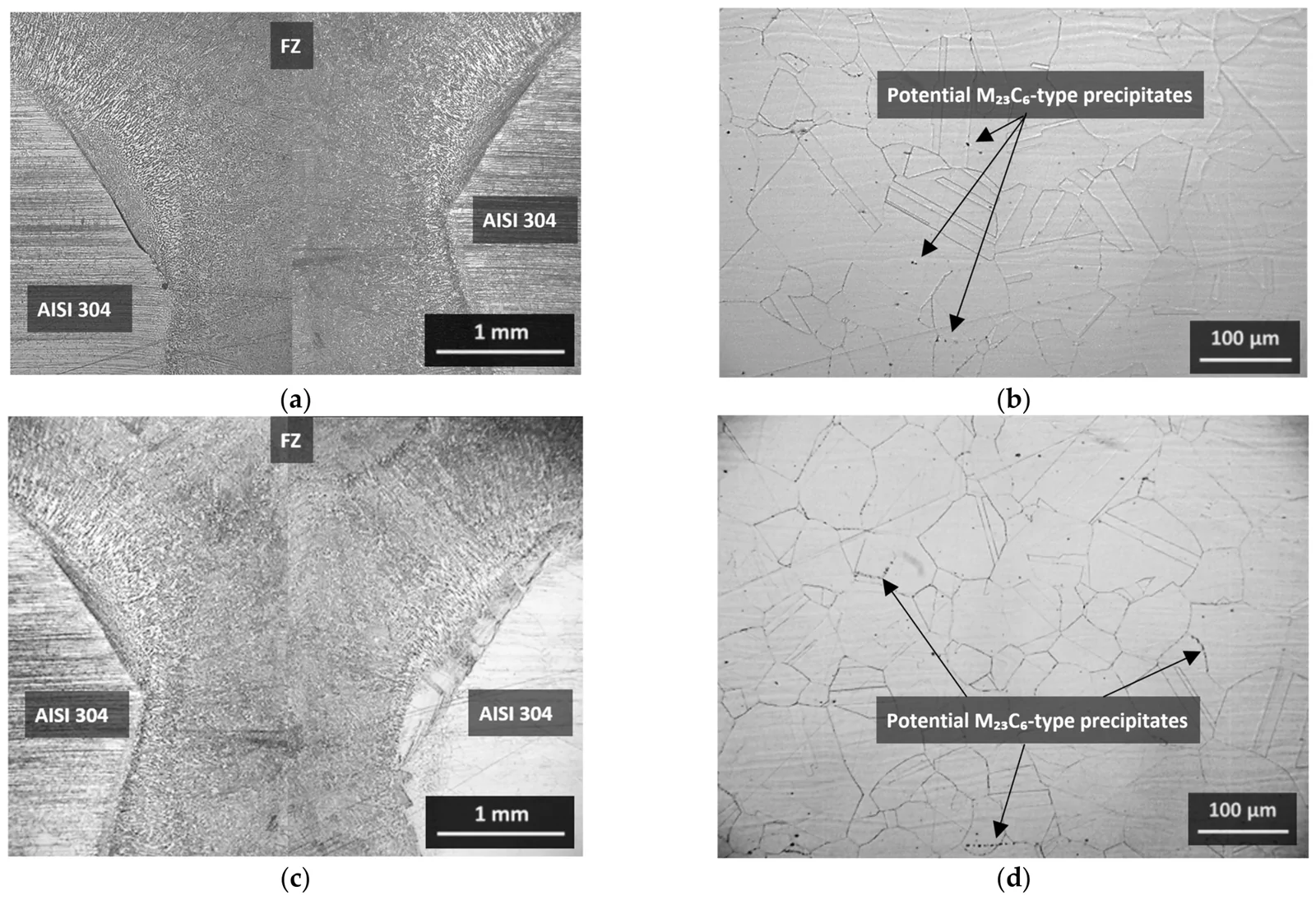
Optical micrographs of the investigated TIG-welded AISI 304 joints: (**a**) general view of the welded-joint microstructure of Specimen 1; (**b**) grain-boundary features in Specimen 1 with morphology consistent with M_23_C_6_-type carbide precipitation; (**c**) corresponding general view of the welded-joint microstructure of Specimen 3; (**d**) corresponding grain-boundary features in Specimen 3. The identification is based on optical morphology and location; no chemical or phase-specific analysis was performed.

**Figure 11:**
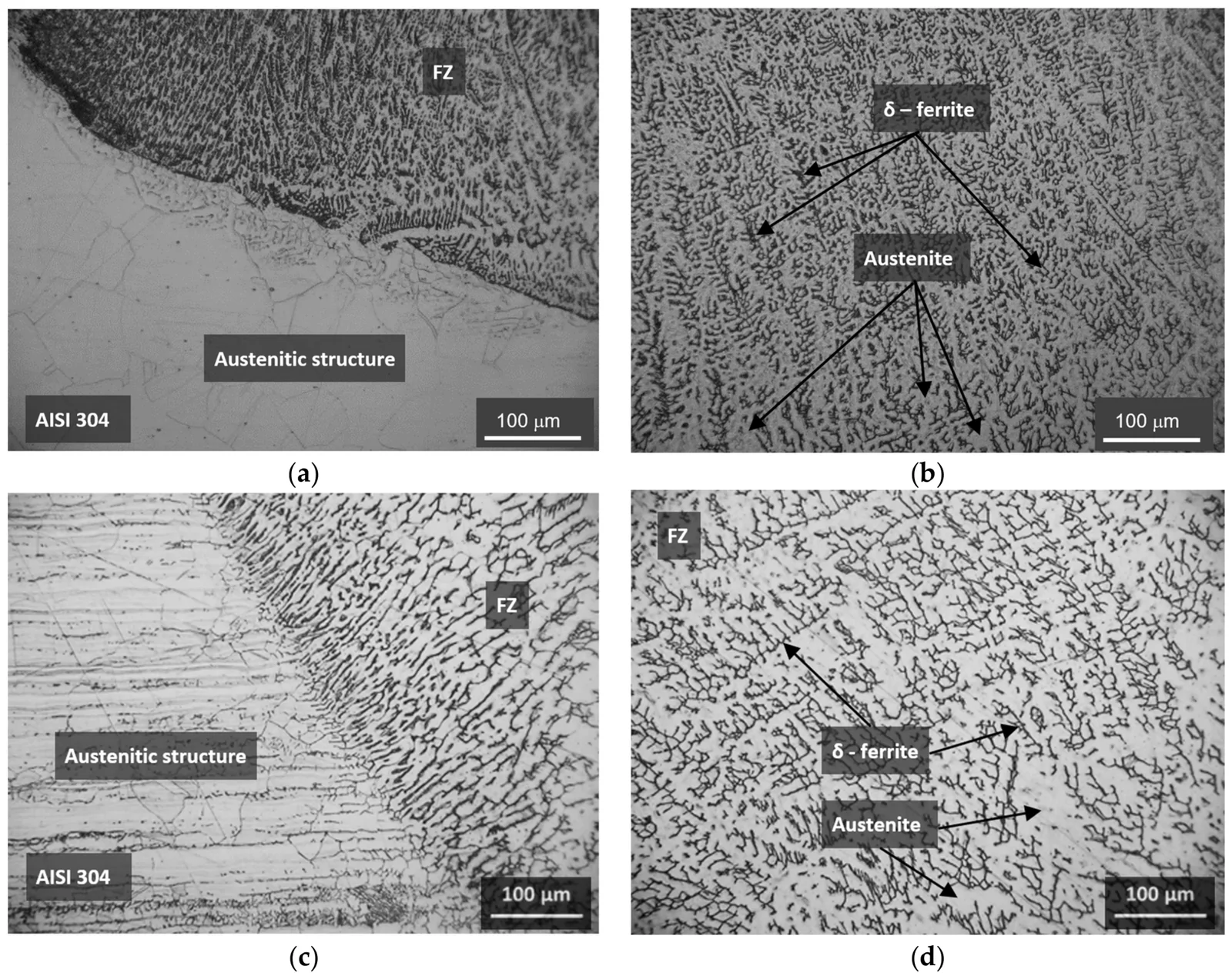
Comparative optical micrographs of the welded regions of Specimens 1 and 3: (**a**) HAZ–FZ interface of Specimen 1; (**b**) dendritic fusion-zone morphology of Specimen 1, with visually identified vermicular and lathy δ-ferrite features within the austenitic weld-metal matrix; (**c**) corresponding HAZ–FZ interface of Specimen 3; (**d**) dendritic fusion-zone morphology of Specimen 3. The micrographs are used for qualitative comparison of the morphology and apparent distribution of the observed microstructural constituents; no quantitative phase-fraction determination was performed.

Microstructural analysis of Specimens 1 and 3 was performed using a Zeiss Axioscope A1 optical microscope (Carl Zeiss Microscopy GmbH, Jena, Germany) equipped with a Zeiss Axiocam 208 color digital imaging camera (Carl Zeiss Microscopy GmbH, Jena, Germany). Optical metallography was used to provide independent qualitative characterization of the microstructural morphology across the base material (BM), heat-affected zone (HAZ), fusion-boundary region, and fusion zone (FZ), and to provide metallurgical context for the macrohardness results. Because these regions experienced different thermal histories during fusion welding, spatial differences in their microstructural morphology were expected [30,53]. Representative optical micrographs of the investigated regions are presented in Figure 10 and Figure 11.

Both welded joints exhibited a clear morphological transition from the thermally affected material toward the fusion zone. The FZ showed the characteristic dendritic solidification morphology associated with melting and subsequent resolidification of the weld metal. The BM exhibited predominantly equiaxed grain morphology typical of wrought austenitic stainless steel. Toward the HAZ and fusion boundary, changes in grain morphology and microstructural appearance became evident, indicating spatial modification of the material structure within the thermally affected region. Such morphological gradients across welded interfaces are consistent with the general metallurgical behaviour reported for fusion-welded metallic materials [30,53].

Within the fusion-zone dendritic matrix, vermicular- and lathy-shaped secondary features characteristic of δ-ferrite in austenitic stainless-steel weld metal were visually identified. Their morphology is consistent with that commonly reported for weld metals produced using 308-type filler materials [30]. The apparent amount and spatial distribution of these features varied locally between the investigated regions and between the two specimens. However, the present evaluation was qualitative; no ferrite-number measurement, image-analysis-based area fraction, or crystallographic phase quantification was performed. Therefore, differences in δ-ferrite content are discussed in terms of visually apparent morphology and distribution rather than as quantitatively measured phase fractions.

Grain-boundary features morphologically consistent with chromium-rich M_23_C_6_-type carbide precipitation were also observed in the thermally affected material, as illustrated in Figure 10b. Such carbide precipitation is metallurgically plausible in austenitic stainless steels subjected to suitable thermal exposure [54]. In the present study, these features were identified visually on the basis of their optical morphology and location along grain boundaries. Their dimensions, number density, chemical composition, and local chromium depletion were not quantitatively determined.

Carbon redistribution and migration have also been reported in welded material systems subjected to strong chemical and thermal gradients [30,55,56]. However, the studies cited in [55,56] concern material systems different from the AISI 304/308LSi joint investigated here and are therefore used only to illustrate possible metallurgical mechanisms.

The comparative observations indicate qualitative differences in the apparent distribution and density of the observed microstructural features between Specimens 1 and 3. Such differences may contribute to variations in the local mechanical response and, consequently, to the spatial dispersion of the measured macrohardness. In particular, differences in dendritic morphology, the visually apparent distribution of δ-ferrite, and grain-boundary precipitation provide plausible metallurgical factors that may contribute to the different hardness-dispersion patterns observed in the two specimens. These observations should nevertheless be interpreted as supporting metallurgical evidence rather than as proof of a direct causal relationship. The present optical analysis does not provide quantitative phase fractions, carbide number densities, grain-size distributions, or local chemical-composition measurements. Consequently, quantitative correlations between individual microstructural parameters and CV(*ν*), WHC(*m*), or tensile properties cannot yet be established. The combination of qualitative metallography, transverse tensile testing, and spatial macrohardness analysis therefore provides complementary evidence for differences between the two welded joints. Future quantitative characterization, including ferrite measurement, digital image analysis, Scanning Electron Microscopy combined with Energy-Dispersive X-ray Spectroscopy (SEM/EDS), Electron Backscatter Diffraction (EBSD), or other suitable phase- and composition-sensitive methods, would allow the visually observed differences to be quantified and correlated more directly with the hardness-dispersion and mechanical-response parameters.

### 3.4. Spatial Variation in Rockwell Macrohardness and Dispersion Descriptors

The spatial distributions of mean Rockwell macrohardness and the corresponding dispersion descriptors were compared between Specimens 1 and 3 at identical measurement positions across the BM-HAZ-FZ-HAZ-BM sequence. Each spatial position corresponds to one measurement line containing 50 individual HRBW observations. The measurement-line designations (−10 to −1 and +1 to +10) are distinguished from their physical positions relative to the weld centerline, which extend from −20 to −2 mm on the left-hand side and from +2 to +20 mm on the right-hand side.

As shown in Table 5, the mean macrohardness profiles of the two specimens were comparatively similar. Specimen 1 exhibited mean values from 92.04 to 95.70 HRBW, whereas Specimen 3 exhibited values from 92.00 to 95.50 HRBW. Meanwhile, substantially greater differences between the specimens became apparent when the dispersion of the 50 measurements at each spatial position was evaluated. For Specimen 1, CV(*ν*) ranged from 0.534% to 1.433%, while WHC(*m*) ranged from 81.20 to 216.95. For Specimen 3, CV(*ν*) ranged from 0.542% to 1.076%, and WHC(*m*) ranged from 107.87 to 214.12. Importantly, the spatial location of the greatest estimated dispersion also differed between the two specimens. In Specimen 1, the maximum CV and minimum WHC occurred within the HAZ at line +4 (+8 mm), whereas in Specimen 3 the greatest point estimates of dispersion occurred within the FZ at line +2 (+4 mm).

These differences indicate that the two welded joints, despite having broadly similar mean-hardness profiles, exhibited distinctly different spatial distributions of local macrohardness dispersion.

The direct comparison in Table 6 reveals substantially different spatial patterns of macrohardness dispersion between the two welded joints. The outer BM regions exhibited closely comparable dispersion levels in Specimens 1 and 3. When averaged descriptively over the six BM measurement positions, CV values were 0.719% and 0.726%, respectively, while the corresponding mean WHC values were 167.43 and 165.44. Thus, the outer BM provided a broadly similar internal dispersion reference in the two specimens.

Differences became more pronounced within the welded and thermally affected regions. In the FZ, the descriptive mean CV increased from 0.930% in Specimen 3 to 1.065% in Specimen 1, while the corresponding mean WHC decreased from 127.10 to 111.39. The largest contrast occurred within the HAZ. The eight HAZ measurement positions yielded a mean CV of 1.182% for Specimen 1 compared with 0.801% for Specimen 3, while the corresponding mean WHC values were 99.80 and 150.02, respectively.

The greatest dispersion point estimate in Specimen 1 occurred at +8 mm within the HAZ, where CV reached 1.433% and WHC decreased to 81.20. A second pronounced local maximum in dispersion was observed at −8 mm, where CV reached 1.325% and WHC decreased to 87.92. In contrast, the greatest dispersion point estimate in Specimen 3 occurred within the FZ at +4 mm, where CV reached 1.076% and WHC decreased to 107.87.

Particularly pronounced local contrasts between the specimens were observed at several HAZ positions. However, because each welding condition is represented by only one mapped specimen, these differences describe the two investigated cross-sections and should not be interpreted as population-level statistically significant effects of welding condition.

Overall, the comparison demonstrates that Specimens 1 and 3 exhibited similar outer-BM macrohardness dispersion but distinctly different dispersion patterns within the welded and thermally affected regions. This spatial contrast was substantially more evident in CV(*ν*) and WHC(*m*) than in the corresponding mean HRBW profiles.

Specimen 1 represents the welded joint exhibiting the poorer tensile response and fracture through the weld region, whereas Specimen 3 represents the joint exhibiting the more favourable tensile response and fracture in the base material. Each point represents the mean of 50 HRBW measurements obtained at the corresponding spatial position; error bars indicate the 95% confidence interval of the line-specific mean. The dashed horizontal reference lines indicate the overall minimum mean hardness (92.00 HRBW) and the maximum mean values observed on the left- and right-hand sides of the investigated cross-sections (95.70 and 95.44 HRBW, respectively).

**Figure 12:**
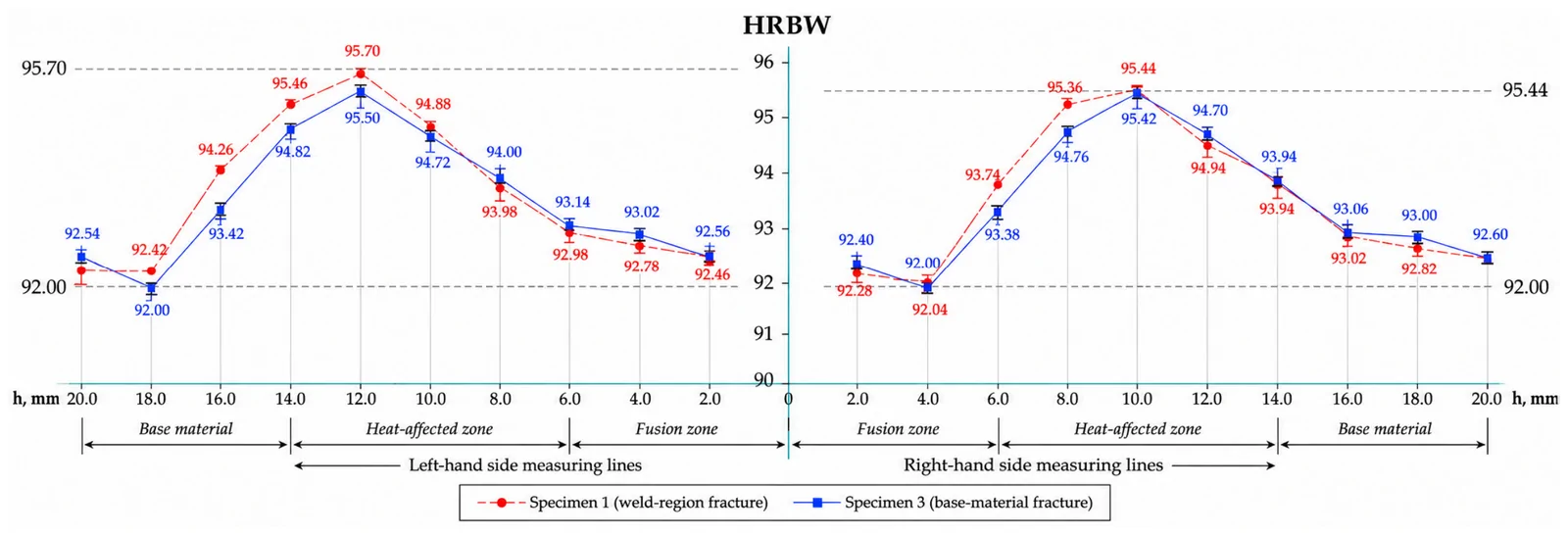
Spatial variation in mean Rockwell macrohardness (HRBW) across Specimens 1 and 3.

Figure 12 compares the spatial distributions of mean Rockwell macrohardness in Specimens 1 and 3. Despite the markedly different tensile responses and fracture locations of the two welded joints, their mean HRBW profiles were generally similar. The mean hardness ranged from 92.04 to 95.70 HRBW in Specimen 1 and from 92.00 to 95.50 HRBW in Specimen 3.

In both specimens, the highest mean hardness values were located within the HAZ at approximately 10 mm from the weld centerline. On the left-hand side, the maximum values were 95.70 HRBW for Specimen 1 and 95.50 HRBW for Specimen 3, whereas on the right-hand side the corresponding maximum values were 95.44 and 95.42 HRBW, respectively. The outer BM regions also exhibited closely comparable mean hardness values. The largest local difference between the two specimens was observed at 6 mm on the left-hand side within the FZ, where the mean hardness was 94.26 HRBW for Specimen 1 and 93.42 HRBW for Specimen 3, corresponding to a difference of 0.84 HRBW.

Overall, the conventional mean-hardness profiles provide only limited discrimination between the two investigated welded joints. This is particularly notable because Specimen 1 exhibited substantially lower tensile strength and elongation and fractured through the weld region, whereas Specimen 3 exhibited the highest tensile response and fractured in the base material. Consequently, the dispersion-based parameters CV(*ν*) and WHC(*m*) were subsequently compared to determine whether the statistical distribution of repeated hardness measurements provides additional discriminatory information beyond the mean HRBW values alone.

Specimen 1 represents the welded joint exhibiting the poorer tensile response and fracture through the weld region, whereas Specimen 3 represents the joint exhibiting the more favourable tensile response and fracture in the base material. Each CV value was calculated from 50 HRBW measurements obtained at the corresponding spatial position. Error bars represent 95% percentile-bootstrap confidence intervals based on 20,000 resamples. Higher CV values indicate greater relative macrohardness dispersion. Specimen 1 is shown by the dashed line and Specimen 3 by the solid line.

**Figure 13:**
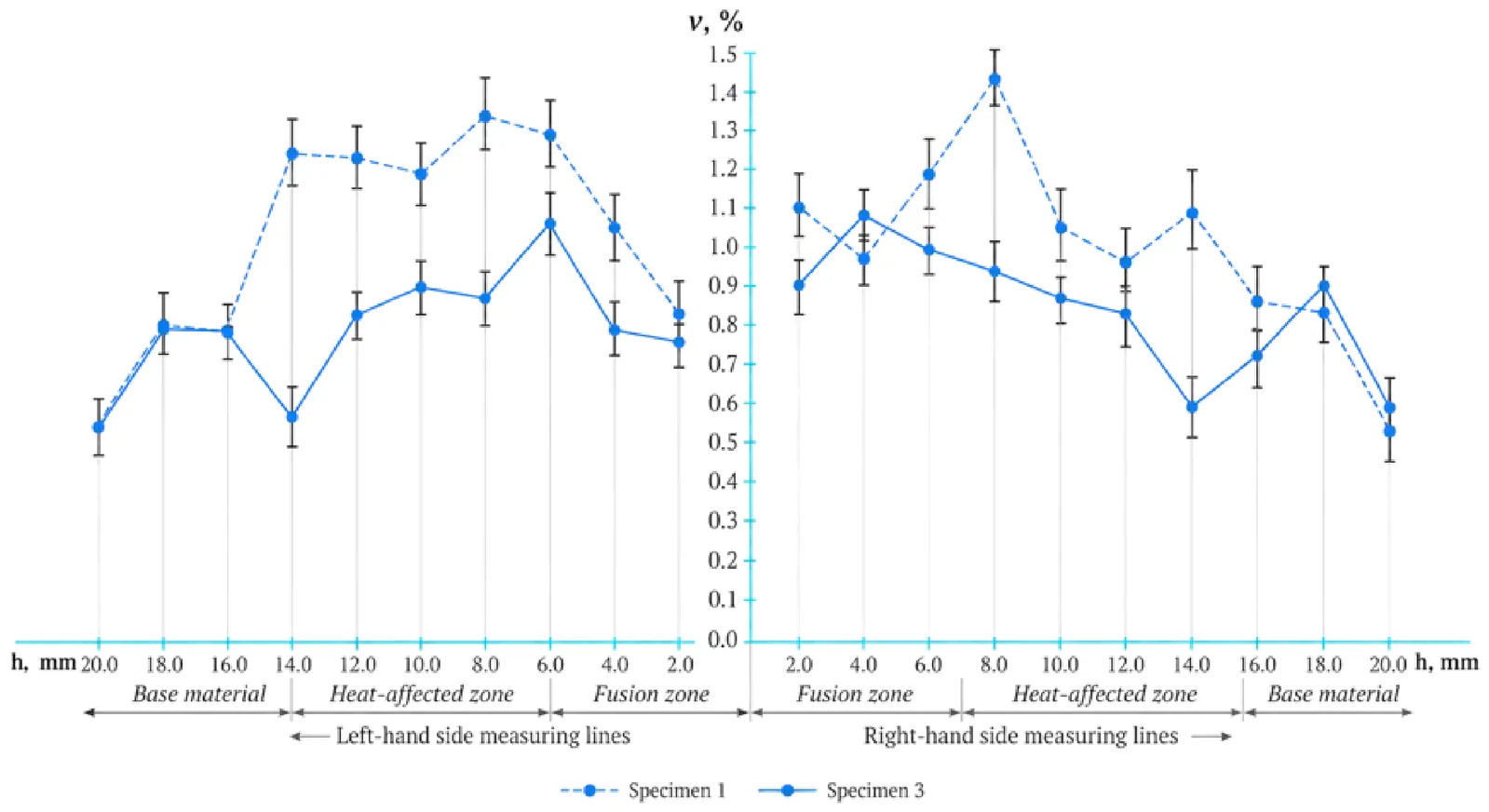
Spatial variation in the coefficient of variation, CV(*ν*), across Specimens 1 and 3.

Figure 13 shows that the spatial behaviour of CV(*ν*) differed considerably more between Specimens 1 and 3 than did their corresponding mean-HRBW profiles. In the outer BM, the CV values of the two specimens remained broadly comparable. Averaged descriptively over the six BM measurement positions, CV was 0.719% for Specimen 1 and 0.726% for Specimen 3. This close agreement indicates that the greater dispersion observed in Specimen 1 was not uniformly distributed throughout the entire cross-section. The differences became more apparent within the FZ and particularly within the HAZ. In the FZ, the descriptively averaged CV increased from 0.930% in Specimen 3 to 1.065% in Specimen 1. A substantially larger difference was observed in the HAZ, where the corresponding mean CV values were 0.801% and 1.182%, respectively. Thus, the average relative hardness dispersion within the HAZ of Specimen 1 was approximately 48% greater than that observed in Specimen 3.

The highest CV point estimate in Specimen 1 was 1.433% at +8 mm from the weld centerline within the HAZ. A second pronounced maximum of 1.325% occurred at the corresponding −8 mm position. In contrast, the maximum CV in Specimen 3 was 1.076% and occurred at +4 mm from the weld centerline within the FZ.

These results demonstrate that Specimen 1, which exhibited the poorer tensile response and fracture through the weld region, also exhibited greater local macrohardness dispersion within the welded and thermally affected regions. Importantly, this contrast was much less evident from the mean-HRBW profiles shown in Figure 12. CV(*ν*) therefore provides additional spatial discrimination between the two investigated welded joints beyond that obtained from conventional comparison of mean hardness alone. Because each welding condition is represented by one mapped specimen, however, the observed differences are interpreted as characteristics of the two investigated cases rather than as population-level effects of welding condition.

Specimen 1 represents the welded joint exhibiting the poorer tensile response and fracture through the weld region, whereas Specimen 3 represents the joint exhibiting the more favourable tensile response and fracture in the base material. Each WHC value was calculated from 50 HRBW measurements obtained at the corresponding spatial position. Error bars represent 95% percentile-bootstrap confidence intervals based on 20,000 resamples. Lower WHC values correspond to greater local macrohardness dispersion, whereas higher WHC values indicate a comparatively narrower local hardness distribution. Specimen 1 is shown by the dashed line and Specimen 3 by the solid line.

**Figure 14:**
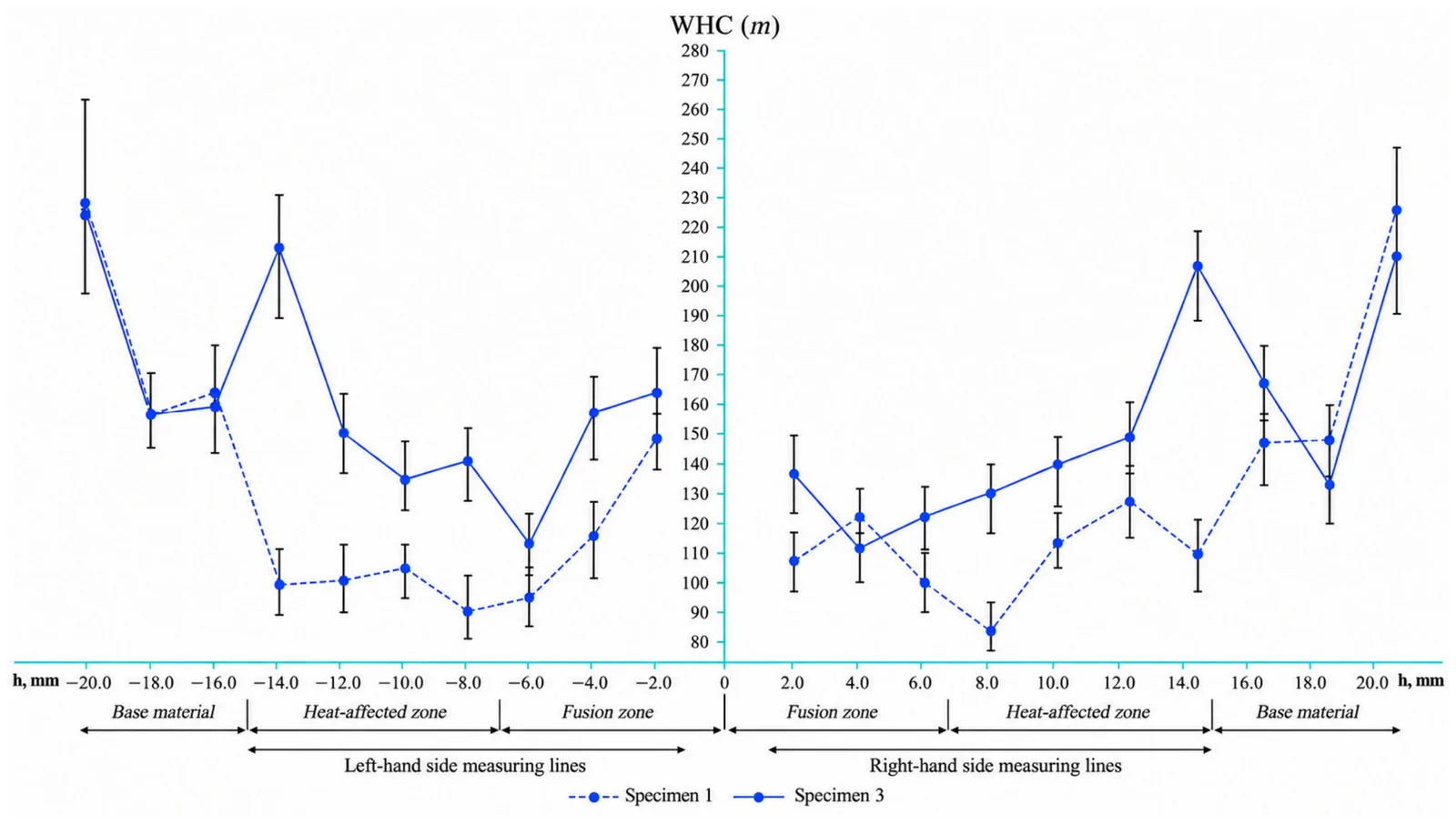
Spatial variation in the Weibull homogeneity coefficient WHC(*m*) across Specimens 1 and 3.

Figure 14 shows that the spatial behaviour of WHC(*m*) was consistent with the corresponding CV(*ν*) trends presented in Figure 13. Positions characterized by higher CV generally exhibited lower WHC, reflecting the inverse relationship between the two dispersion descriptors derived from the same local HRBW datasets.

The difference between Specimens 1 and 3 was most pronounced within the HAZ. In Specimen 1, WHC decreased to 87.92 on the left-hand side and to 81.20 on the right-hand side at 8 mm from the weld centerline. At the corresponding positions, Specimen 3 exhibited substantially higher WHC values of 133.78 and 123.26, respectively. When averaged descriptively over the eight HAZ measurement positions, WHC was 99.80 for Specimen 1 and 150.02 for Specimen 3.

A similar, although less pronounced, tendency was observed within the FZ. The descriptively averaged WHC was 111.39 for Specimen 1 and 127.10 for Specimen 3. In contrast, the outer BM regions of the two specimens showed very similar values, with average WHC values of 167.43 and 165.44, respectively. Thus, the lower WHC observed in Specimen 1 was concentrated primarily within the welded and thermally affected regions rather than representing uniformly greater dispersion throughout the entire cross-section.

Taken together, Figure 13 and Figure 14 show that the joint exhibiting the poorer tensile response and fracture through the weld region also exhibited greater macrohardness dispersion, expressed by higher CV and lower WHC, particularly within the HAZ. This contrast was substantially more evident in the dispersion-based descriptors than in the corresponding mean HRBW profiles. The results therefore support the use of spatial CV and WHC mapping as complementary descriptors for differentiating welded joints with contrasting mechanical responses. However, because each welding condition is represented by a single mapped specimen, the observed relationship should be interpreted as a case-specific association rather than as a generally validated predictive relationship between macrohardness dispersion and tensile performance.

The sample-size sensitivity analysis showed the same general behaviour in both investigated specimens (Table 7). The stability of CV(*ν*) and WHC(*m*) progressively improved as the number of hardness measurements included in the calculation increased. For Specimen 1, the median absolute relative deviation of CV decreased from 8.36% at *n* = 15 to 2.64% at *n* = 40, while the corresponding WHC deviation decreased from 13.82% to 3.09%. Similar behaviour was observed for Specimen 3, for which the median deviations decreased from 9.74% to 3.11% for CV and from 13.79% to 3.47% for WHC.

The 95th-percentile deviations showed the same tendency. At *n* = 15, deviations approaching approximately 28–31% occurred for both dispersion descriptors, whereas at *n* = 40 the 95th-percentile deviations decreased to below approximately 10% in both specimens. WHC generally showed greater sensitivity than CV when relatively small subsets were used, particularly at *n* = 15–20.

The agreement of the sensitivity trends between Specimens 1 and 3 indicates that the observed improvement in estimator stability with increasing sampling density was not restricted to a single welded joint or to one particular level of macrohardness dispersion. The results support the use of the complete set of 50 measurements per line for the present comparative analysis. However, they do not establish n = 50 as a universal minimum sampling requirement, because the required number of measurements may depend on the material, hardness scale, surface condition, spatial variability, and required statistical precision.

## 4. Discussion

The direct comparison of Specimens 1 and 3 demonstrates an important distinction between the spatial variation in mean Rockwell macrohardness and the statistical dispersion of repeated hardness measurements. Although the two welded joints exhibited markedly different tensile responses and fracture locations, their mean HRBW profiles remained comparatively similar. Mean hardness varied from 92.04 to 95.70 HRBW in Specimen 1 and from 92.00 to 95.50 HRBW in Specimen 3. Both specimens exhibited the same general spatial tendency, with lower mean hardness predominantly within the FZ and higher values within parts of the HAZ. The outer BM values were also closely comparable. Consequently, evaluation based on mean hardness alone provided relatively limited discrimination between the two investigated welding conditions.

A substantially clearer distinction emerged when the distributions of repeated hardness measurements were evaluated using CV(*ν*) and WHC(*m*). Specimen 1, which exhibited the lowest UTS (610 MPa), the lowest elongation (24%), and fracture through the weld region, generally showed higher CV and lower WHC within the welded and thermally affected regions than Specimen 3, which exhibited the highest UTS (684 MPa), 41% elongation, and fracture in the base material. This difference was particularly pronounced within the HAZ. The descriptively averaged CV across the HAZ increased from 0.801% in Specimen 3 to 1.182% in Specimen 1, while the corresponding mean WHC decreased from 150.02 to 99.80.

The contrasting HAZ behaviour is particularly relevant because the outer BM regions of the two specimens exhibited almost identical dispersion characteristics. The mean CV values calculated across the BM measurement positions were 0.719% for Specimen 1 and 0.726% for Specimen 3, while the corresponding mean WHC values were 167.43 and 165.44, respectively. Specimen 1 therefore cannot be described simply as exhibiting uniformly greater hardness scatter throughout the entire cross-section. Instead, the increased dispersion was spatially concentrated within the weld-affected regions, particularly the HAZ. This localization provides substantially stronger discrimination between the two investigated joints than the absolute hardness level alone.

Differences were also observed within the FZ, although they were less pronounced than those within the HAZ. The descriptively averaged CV in the FZ was 1.065% for Specimen 1 and 0.930% for Specimen 3, while the corresponding mean WHC values were 111.39 and 127.10. The spatial locations of the greatest dispersion estimates also differed between the specimens. In Specimen 1, the lowest WHC values occurred within the HAZ, reaching 87.92 and 81.20 at positions 8 mm from the weld centerline on the left- and right-hand sides, respectively. In Specimen 3, the greatest macrohardness-dispersion point estimate occurred within the FZ, where CV reached 1.076% and WHC decreased to 107.87 at +4 mm. Thus, the two specimens differed not only in the magnitude of local macrohardness dispersion but also in its spatial distribution across the welded joint.

These results provide experimental support for the principal methodological concept investigated in the present study: statistical analysis of repeated macrohardness measurements can reveal differences between welded joints that are considerably less evident from conventional mean-hardness profiles. The additional information does not arise from a new hardness scale or from newly introduced statistical parameters. CV(*ν*) and WHC(*m*) are established dispersion descriptors [6,39,40]. The methodological contribution of the present work lies in combining these descriptors with dense spatial Rockwell macrohardness mapping and applying the same measurement strategy at corresponding positions in two welded joints exhibiting distinctly different mechanical responses.

This direct comparison also provides a stronger evaluation of the practical discriminatory capability of the spatial macrohardness methodology than analysis of a single welded specimen. The outer BM behaviour of Specimens 1 and 3 was very similar in terms of both mean hardness and hardness dispersion, whereas substantial differences appeared when approaching the welded and thermally affected regions. Therefore, the differentiation achieved using CV and WHC cannot be attributed merely to a general shift in hardness level between the two specimens. Rather, the dispersion-based parameters reveal localized differences in the distribution of repeated hardness measurements within specific regions of the welded joints.

The tensile-test results provide an independent mechanical context for this comparison. The specimen exhibiting greater hardness dispersion within the weld-affected regions was also the specimen that showed the poorer tensile response and fractured through the weld region. Conversely, Specimen 3 exhibited lower macrohardness dispersion, particularly within the HAZ, together with the more favourable tensile response and fracture in the base material. For the two investigated cases, this correspondence supports the working hypothesis that spatial analysis of hardness dispersion can provide discriminatory information that is not evident from mean hardness alone.

Nevertheless, the present results do not demonstrate that CV(*ν*) or WHC(*m*) can quantitatively predict tensile strength, elongation, or fracture location. Specimens 1 and 3 represent different welding conditions, and only one welded specimen from each condition was subjected to detailed spatial macrohardness mapping. The observed correspondence between greater macrohardness dispersion and the poorer tensile response must therefore be interpreted as an association between two contrasting investigated cases rather than as a generally validated predictive relationship. Accordingly, the observed differences in CV(*ν*) and WHC(*m*) apply only to the two specific experimental conditions investigated here and do not demonstrate a general correlation between macrohardness dispersion and mechanical strength. Confirmation of such a relationship would require independently produced replicate welds under each condition and direct statistical evaluation of relationships between hardness-dispersion parameters and mechanical properties.

The welding conditions applied to Specimens 1 and 3 resulted in substantially different calculated heat inputs. Specimen 1 was welded using calculated heat inputs of 169.71 J/mm for the root pass and 158.40 J/mm for the hot, fill, and cap passes, whereas the corresponding values for Specimen 3 were 264.00 and 218.40 J/mm. Heat input is known to influence the thermal history, solidification conditions, and microstructural evolution of welded materials [15,16,17,18,19,20,21,22,23,24,30]. The differences in welding conditions are therefore metallurgically relevant when interpreting the contrasting behaviour of the two investigated joints.

However, complete welding thermal cycles and cooling rates were not directly measured in the present experiments. Consequently, the observed differences in tensile response, microstructural morphology, CV(*ν*), and WHC(*m*) cannot be quantitatively attributed to heat input or cooling rate. The calculated heat-input values characterize the imposed welding conditions but do not represent direct measurements of the local thermal histories experienced at individual positions across the welded joints.

Optical metallography provides complementary qualitative microstructural context for the mechanical and macrohardness results. Both specimens exhibited a clear transition from the thermally affected material toward a dendritic fusion-zone structure. Within the FZ, vermicular- and lathy-shaped features morphologically characteristic of δ-ferrite in austenitic stainless-steel weld metal were visually identified. Similar morphologies have been reported for austenitic stainless-steel weld metals produced using 308-type filler materials [30]. Differences in the visually apparent morphology and spatial distribution of these features were observed between the investigated specimens. However, no ferrite-number measurement, quantitative image analysis, or crystallographic phase identification was performed; therefore, differences in δ-ferrite content cannot be quantified from the present optical observations.

Grain-boundary features morphologically consistent with M_23_C_6_-type carbide precipitation were also observed in the thermally affected material. Formation of M_23_C_6_ carbides at austenite grain boundaries under suitable thermal exposure has been reported for austenitic stainless steels and may be associated with local chromium depletion [54]. In the present study, however, these features were identified solely from their optical morphology and grain-boundary location. Their chemical composition, dimensions, number density, and any associated chromium depletion were not measured. Accordingly, their interpretation as M_23_C_6_-type precipitates should be regarded as metallurgically plausible rather than analytically confirmed.

Carbon redistribution and migration have also been reported in welded material systems subjected to suitable chemical and thermal gradients [30,55,56]. Because the material systems investigated in [55,56] differ from the AISI 304/308LSi joint examined here, these studies are cited only as examples of possible metallurgical mechanisms and not as direct evidence that carbon migration occurred in the present specimens.

The metallographic interpretation therefore remains qualitative. No quantitative δ-ferrite fraction, ferrite number, carbide number density, grain-size distribution, local chemical composition, or chromium-depletion profile was determined. Consequently, the observed microstructural differences cannot presently be correlated quantitatively with CV(*ν*), WHC(*m*), or the tensile properties. The optical observations provide complementary metallurgical context for the differences between Specimens 1 and 3 rather than direct proof of the mechanisms responsible for the measured macrohardness dispersion. Quantitative image analysis, ferrite measurement, SEM/EDS, EBSD, XRD, or other suitable phase- and composition-sensitive methods would be required to establish such relationships.

An important distinction also concerns the interpretation of WHC(*m*). Previous MHC studies have used *m* = 10 as a practical boundary for distinguishing structurally heterogeneous and homogeneous material states [6,49]. However, an absolute material-independent threshold cannot be assumed without validation for a specific alloy, welding process, hardness scale, sampling strategy, and microstructural condition. In the present investigation, all WHC values were substantially above this previously proposed threshold. The minimum WHC value was 81.20 in Specimen 1 and 107.87 in Specimen 3. Consequently, the *m* = 10 criterion does not provide meaningful discrimination between either the investigated weld regions or the two welded joints.

WHC(*m*) is therefore interpreted in the present study as a continuous comparative descriptor of macrohardness dispersion rather than as an absolute homogeneous/heterogeneous classification criterion. Lower WHC values correspond to greater dispersion of the logarithmically transformed local hardness distribution, whereas higher values correspond to a comparatively narrower distribution [39,40]. This approach allows relative spatial differences to be evaluated without assigning an experimentally unvalidated structural classification to the investigated material.

The inverse spatial behaviour of CV(*ν*) and WHC(*m*) is expected because both parameters are calculated from the same underlying HRBW datasets and characterize dispersion using related mathematical formulations [6,39,40]. Their agreement should therefore not be interpreted as independent validation of a physical microstructural state. CV describes relative scatter on the original hardness scale, whereas WHC characterizes dispersion of the logarithmically transformed hardness measurements. Their value in the present analysis lies in providing complementary numerical representations of the same underlying local macrohardness variability and in demonstrating consistent spatial trends between the two investigated joints.

The complementary roles of the experimental and statistical methods used in the present investigation are summarized in Table 8.

The methods summarized in Table 8 provide complementary rather than interchangeable information. Transverse tensile testing characterizes the global mechanical response and fracture location of the welded joints, optical metallography provides qualitative information on local microstructural morphology, and Rockwell testing provides spatially resolved indentation-hardness measurements. Statistical evaluation of repeated HRBW measurements using CV(*ν*) and WHC(*m*) adds information on the spatial distribution of local hardness dispersion. The direct comparison of Specimens 1 and 3 demonstrates that this dispersion-based information can differentiate two welded joints whose mean-hardness profiles are comparatively similar but whose tensile responses and fracture locations are markedly different. Nevertheless, the present results do not establish equivalence with, superiority over, or replacement of tensile testing, quantitative metallography, or established non-destructive testing methods.

The sample-size sensitivity analysis further demonstrated that the stability of both dispersion descriptors depends on the number of hardness measurements included in the calculation. The same general trend was observed for both investigated specimens. For Specimen 1, the median absolute relative deviation of CV decreased from 8.36% at *n* = 15 to 2.64% at *n* = 40, while the corresponding deviation of WHC decreased from 13.82% to 3.09%. For Specimen 3, the median CV deviation decreased from 9.74% to 3.11%, while the WHC deviation decreased from 13.79% to 3.47% over the same range of sample sizes.

The corresponding 95th-percentile deviations showed the same tendency. At *n* = 15, deviations approaching approximately 28–31% occurred for the dispersion descriptors, whereas at *n* = 40 the 95th-percentile deviations were below approximately 10% for both specimens. The broadly similar sensitivity behaviour of Specimens 1 and 3 indicates that the progressive increase in estimator stability with sampling density was not restricted to one particular welded joint or one specific level of macrohardness dispersion. These results support the use of all 50 measurements per line in the present analysis but do not establish *n* = 50 as a universal minimum sampling requirement. The number of measurements required in another application will depend on the material, hardness scale, surface condition, local variability, and required statistical precision.

The experimental replication structure remains an important limitation of the present study. Although the revised analysis includes two welded specimens with distinctly different mechanical responses, Specimens 1 and 3 were produced using different welding conditions and are not replicate welds. Each specimen contains 20 spatial measurement lines with 50 local HRBW measurements per line, resulting in 1000 measurements per specimen and 2000 measurements in the comparative dataset. These measurements provide high-resolution spatial information but do not constitute 2000 independent welded-joint replicates.

Accordingly, the reported confidence intervals characterize uncertainty associated with the local hardness measurements and the corresponding statistical estimators. They do not quantify between-weld reproducibility. Differences between Specimens 1 and 3 should therefore not be interpreted as population-level statistically significant effects of welding condition. Independently produced replicate welds under each investigated condition would be required for confirmatory inferential testing, quantitative assessment of between-weld reproducibility, and generalization of the observed relationship between hardness dispersion and mechanical response.

The outer BM regions provide useful internal reference areas for the comparative analysis because both specimens exhibited closely similar mean hardness and dispersion descriptors in these regions. This bilateral and between-specimen agreement strengthens the interpretation that the more pronounced differences observed within the HAZ and FZ are spatially associated with the weld-affected regions rather than with a uniform difference between the two specimens. Nevertheless, no independent pre-weld hardness maps or quantitative pre-weld microstructural measurements were available. The contribution of any pre-existing spatial variability in the original plate material therefore cannot be separated completely from changes associated with welding.

A further distinction should be made between the controlled laboratory implementation used in the present study and potential industrial or field application. The experimental procedure included removal of the weld reinforcement by precision milling to provide a sufficiently flat surface for Rockwell testing and employed a deliberately dense measurement grid. Rockwell testing also produces permanent local indentations. The present approach should therefore be regarded as a localized, minimally destructive indentation-based material-characterization procedure rather than as conventional NDT. Any reduction in surface-preparation requirements or sampling density would require separate validation before routine industrial application.

The methodology should also not be interpreted as a comprehensive weld-quality assessment. Weld quality is multidimensional and may involve tensile performance, fracture behaviour, internal and surface defects, weld geometry, residual stresses, corrosion behaviour, and quantitative microstructural characteristics. The macrohardness-based statistical analysis used here directly evaluates only the spatial level and dispersion of Rockwell hardness measurements. Its experimentally supported contribution is therefore the provision of additional spatial information on local material response beyond that available from mean HRBW alone.

Overall, the direct comparison of Specimens 1 and 3 substantially strengthens the experimental evidence for the discriminatory value of spatial macrohardness-dispersion mapping. Two welded joints exhibiting broadly similar mean HRBW profiles showed markedly different spatial distributions of CV and WHC, with the greater differences concentrated particularly within the HAZ. The specimen exhibiting the poorer tensile response and weld-region fracture also exhibited greater local macrohardness dispersion within the weld-affected regions. These findings support further investigation of spatial CV and WHC mapping as complementary descriptors for welded-joint characterization.

Further work should determine whether the observed relationship is reproducible across independently produced replicate welds and whether CV(*ν*) and WHC(*m*) correlate quantitatively with tensile properties, fracture behaviour, grain-size distribution, δ-ferrite fraction, carbide distribution, residual stress, local chemical composition, and other independently measured characteristics. Such validation is required before the hardness-dispersion descriptors can be extended from comparative characterization of individual welded joints toward broader predictive or weld-quality assessment applications.

## 5. Conclusions

The spatial macrohardness analysis of two TIG-welded AISI 304 specimens exhibiting contrasting tensile responses demonstrated that mean Rockwell macrohardness alone provided limited differentiation between the investigated joints. Mean HRBW values ranged from 92.04 to 95.70 in Specimen 1 and from 92.00 to 95.50 in Specimen 3, resulting in broadly similar spatial mean-hardness profiles despite substantial differences in tensile behaviour.

A considerably stronger distinction was obtained from the dispersion-based descriptors CV(*ν*) and WHC(*m*). The largest between-specimen difference was observed within the HAZ. The descriptively averaged CV was 1.182% for Specimen 1 and 0.801% for Specimen 3, whereas the corresponding mean WHC values were 99.80 and 150.02, respectively. Within the FZ, the corresponding average CV values were 1.065% and 0.930%, while the mean WHC values were 111.39 and 127.10. In contrast, the outer BM regions exhibited closely comparable dispersion characteristics, with average CV values of 0.719% and 0.726% and mean WHC values of 167.43 and 165.44 for Specimens 1 and 3, respectively. The greater dispersion observed in Specimen 1 was therefore concentrated primarily within the weld-affected regions rather than representing a uniform increase in hardness variability throughout the specimen.

The macrohardness-dispersion results corresponded to the contrasting tensile behaviour of the two selected specimens. Specimen 1 exhibited the lowest UTS among the five investigated joints (610 MPa), the lowest elongation (24%), and fracture through the weld region, whereas Specimen 3 exhibited the highest UTS (684 MPa), 41% elongation, and fracture in the base material. The specimen exhibiting the poorer tensile response therefore also showed greater local macrohardness dispersion, particularly within the HAZ. This correspondence demonstrates the discriminatory potential of spatial CV and WHC mapping for distinguishing the two investigated welded joints despite their comparatively similar mean-hardness profiles. However, because only one detailed macrohardness map was obtained for each welding condition, this correspondence represents a case-specific association and should not be interpreted as a validated predictive relationship between hardness dispersion, tensile properties, or fracture location.

Optical metallography revealed qualitative differences in the morphology and visually apparent distribution of microstructural features within the BM–HAZ–FZ sequence of the two specimens. Dendritic weld-metal features characteristic of δ-ferrite and grain-boundary features morphologically consistent with M_23_C_6_-type carbide precipitation provided metallurgical context for the observed spatial variations. However, δ-ferrite fraction, carbide density, grain-size distribution, and local chemical composition were not quantitatively determined. Consequently, the present microstructural observations provide supporting qualitative evidence rather than quantitative confirmation of the mechanisms responsible for the observed macrohardness dispersion.

The previously proposed WHC threshold of *m* = 10 did not provide meaningful discrimination for the investigated AISI 304/308LSi welded joints because all calculated WHC values were substantially higher. WHC was therefore applied as a continuous comparative descriptor, with lower values corresponding to greater local macrohardness dispersion and higher values corresponding to a comparatively narrower hardness distribution. Because CV and WHC are derived from the same HRBW datasets, their consistent spatial trends should be regarded as complementary mathematical descriptions of hardness dispersion rather than as independent validation of material homogeneity.

The sample-size sensitivity analysis showed that the stability of both dispersion descriptors improved progressively as the number of hardness measurements per line increased. In both specimens, the median deviations from the complete *n* = 50 estimates decreased substantially as the subset size increased from *n* = 15 to *n* = 40, while the 95th-percentile deviations decreased to below approximately 10% at *n* = 40. These findings support the use of 50 measurements per line for the present experimental configuration but do not establish a universal minimum sampling requirement.

In total, the comparative macrohardness dataset comprised 2000 HRBW measurements obtained from 40 spatial measurement lines across the two selected welded specimens. These measurements provide dense within-specimen spatial information but do not constitute independent welded-joint replication. The present study therefore demonstrates that spatial analysis of repeated Rockwell macrohardness measurements using CV(*ν*) and WHC(*m*) can provide additional discriminatory information beyond conventional mean-hardness profiling for the two investigated contrasting cases. Further studies should include independently replicated welds under each welding condition and quantitative evaluation of relationships between hardness-dispersion descriptors and tensile properties, fracture behaviour, δ-ferrite fraction, grain-size distribution, carbide characteristics, residual stresses, and local chemical composition before broader predictive or weld-assessment applications are considered.

## Figures and Tables

**Table 1:** Chemical composition of the AISI 304 base material and OK Tigrod 308LSi filler rod.

Grade	Chemical Composition [%]									
	C	Mn	Si	P	S	Cr	Ni	Cu	N	Mo
AISI 304	0.027	1.780	0.320	0.030	0.0010	18.05	8.05	-	0.066	-
OK Tigrod 308LSi *	0.010	1.800	0.800	0.020	0.0100	20.00	10.50	0.100	0.110	0.10

Fe—balance; *—filler rod.

**Table 2:** Nominal mechanical properties of the AISI 304 base material and OK Tigrod 308LSi filler rod.

Grade	Mechanical Properties					
	0.2% Proof Strength *R_p_*_0.2_ [MPa]	1.0% Proof Strength *R_p_*_1.0_ [MPa]	Tensile Strength *R_m_* [MPa]	Elongation [%]	Young’s Modulus [GPa]	Hardness [HV]
AISI 304	288	333	609	59.3	190–203	167
OK Tigrod 308LSi *	480	-	625	37	-	189

*—Filler rod.

**Table 3:** Main parameters of the welding procedure.

Specimen No.	Pass	TIG Voltage [V]	TIG Current [A]	Travel Speed [mm/s]	Heat Input [J/mm]
Specimen 1	Root pass	11.0	90	3.5	169.71
	Hot, fill, and cap passes	11.0	120	5.0	158.40
Specimen 2	Root pass	13.0	100	3.5	222.86
	Hot, fill, and cap passes	13.0	125	5.0	195.00
Specimen 3	Root pass	14.0	110	3.5	264.00
	Hot, fill, and cap passes	14.0	130	5.0	218.40
Specimen 4	Root pass	14.5	110	3.5	273.43
	Hot, fill, and cap passes	14.5	140	5.0	243.60
Specimen 5	Root pass	15.0	110	3.5	282.86
	Hot, fill, and cap passes	15.0	150	5.0	270.00

TIG thermal efficiency factor, *η* = 0.6. The arc length was maintained at approximately 3 mm during welding.

**Table 4:** Tensile test results of the welded AISI 304 specimens.

Specimen No.	UTS (MPa)	Elongation (%)	Fracture Location
1	610	24	Weld region
2	641	31	Base material
3	684	41	Base material
4	670	38	Base material
5	661	35	Base material

**Table 5:** Comparison of mean Rockwell macrohardness values measured at corresponding spatial positions in Specimens 1 and 3.

Measuring Line	Zone	Distance from the Weld Centerline (mm)	Specimen 1 Mean Hardness (HRBW)	Specimen 3 Mean Hardness (HRBW)
Left side				
−10	BM	−20	92.46	92.56
−9	BM	−18	92.78	93.02
−8	BM	−16	92.98	93.14
−7	HAZ	−14	93.84	94.00
−6	HAZ	−12	94.88	94.72
−5	HAZ	−10	95.70	95.50
−4	HAZ	−8	95.46	94.82
−3	FZ	−6	94.26	93.42
−2	FZ	−4	92.42	92.00
−1	FZ	−2	92.30	92.54
Right side				
+1	FZ	+2	92.28	92.40
+2	FZ	+4	92.04	92.00
+3	FZ	+6	93.74	93.38
+4	HAZ	+8	95.36	94.76
+5	HAZ	+10	95.44	95.42
+6	HAZ	+12	94.62	94.70
+7	HAZ	+14	93.94	93.94
+8	BM	+16	93.02	93.06
+9	BM	+18	92.82	93.00
+10	BM	+20	92.60	92.60

**Table 6:** Spatial variation and statistical uncertainty of Rockwell macrohardness and dispersion descriptors across the investigated welded specimens.

Line No.	Distance [mm]	Zone	Specimen 1 CV, % [95% *CI*]	Specimen 3 CV, % [95% *CI*]	Specimen 1 WHC [95% *CI*]	Specimen 3 WHC [95% *CI*]
−10	−20	BM	0.545 [0.510–0.546]	0.542 [0.499–0.546]	213.25 [212.57–227.85]	214.12 [212.57–231.93]
−9	−18	BM	0.794 [0.653–0.914]	0.798 [0.646–0.933]	146.24 [126.94–177.77]	145.30 [124.20–179.56]
−8	−16	BM	0.768 [0.646–0.860]	0.782 [0.660–0.875]	151.16 [135.05–179.56]	148.22 [132.57–175.83]
−7	−14	HAZ	1.225 [0.995–1.408]	0.569 [0.428–0.680]	94.99 [82.56–116.92]	204.11 [170.77–271.68]
−6	−12	HAZ	1.217 [1.008–1.381]	0.827 [0.689–0.937]	95.54 [84.08–115.26]	140.47 [123.91–168.58]
−5	−10	HAZ	1.180 [0.998–1.329]	0.904 [0.739–1.035]	98.45 [87.35–116.42]	128.53 [112.09–157.19]
−4	−8	HAZ	1.325 [1.077–1.514]	0.870 [0.684–1.022]	87.92 [76.94–108.13]	133.78 [114.08–170.13]
−3	−6	FZ	1.283 [1.104–1.427]	1.061 [0.892–1.193]	90.51 [81.36–105.33]	109.47 [97.51–130.26]
−2	−4	FZ	1.050 [0.851–1.220]	0.792 [0.598–0.959]	110.50 [95.04–136.49]	146.58 [121.08–193.59]
−1	−2	FZ	0.826 [0.657–0.969]	0.763 [0.621–0.893]	140.62 [119.93–176.63]	152.26 [130.05–187.27]
+1	+2	FZ	1.096 [0.887–1.278]	0.902 [0.731–1.040]	106.22 [91.11–130.99]	128.86 [111.52–158.86]
+2	+4	FZ	0.956 [0.759–1.126]	1.076 [0.836–1.271]	121.59 [103.25–153.28]	107.87 [91.23–138.85]
+3	+6	FZ	1.177 [0.953–1.351]	0.989 [0.815–1.127]	98.88 [85.97–121.97]	117.53 [103.05–142.61]
+4	+8	HAZ	1.433 [1.174–1.635]	0.943 [0.732–1.119]	81.20 [71.14–99.20]	123.26 [103.81–158.81]
+5	+10	HAZ	1.040 [0.797–1.254]	0.875 [0.709–1.011]	111.49 [92.58–145.82]	132.68 [114.94–163.76]
+6	+12	HAZ	0.952 [0.748–1.134]	0.833 [0.639–1.030]	121.67 [102.02–155.25]	139.06 [112.08–181.60]
+7	+14	HAZ	1.084 [0.858–1.270]	0.585 [0.450–0.694]	107.12 [91.41–135.31]	198.23 [167.30–257.62]
+8	+16	BM	0.841 [0.728–0.922]	0.733 [0.608–0.836]	137.95 [125.93–159.22]	158.23 [138.73–190.99]
+9	+18	BM	0.834 [0.675–0.974]	0.922 [0.762–1.053]	139.00 [118.96–171.91]	125.70 [109.93–152.10]
+10	+20	BM	0.534 [0.478–0.546]	0.577 [0.508–0.655]	216.95 [212.57–242.31]	201.07 [177.58–227.85]

Note: *n* = 50 HRBW measurements per line and specimen. Bracketed intervals represent 95% percentile-bootstrap confidence intervals based on 20,000 resamples. Specimens 1 and 3 represent different welding conditions and are not replicate welds.

**Table 7:** Sensitivity of CV(*ν*) and WHC(*m*) to the number of hardness measurements per line.

*n*	*d*(*n*)	Median Absolute Relative Deviation of CV from *n* = 50, %	95th Percentile Deviation of CV, %	Median Absolute Relative Deviation of WHC from *n* = 50, %	Percentile Deviation of WHC, %
Specimen 1					
15	1.0206	8.36	27.61	13.82	29.15
20	1.0628	6.75	21.94	9.85	22.94
25	1.0915	5.45	17.54	7.35	18.56
30	1.1124	4.37	14.18	5.62	14.72
40	1.1413	2.64	8.62	3.09	8.65
50	1.1607	Reference	Reference	Reference	Reference
Specimen 3					
15	1.0206	9.74	29.38	13.79	30.76
20	1.0628	7.44	23.70	10.32	24.32
25	1.0915	6.33	18.88	7.78	19.31
30	1.1124	5.04	15.61	6.38	15.88
40	1.1413	3.11	9.35	3.47	9.27
50	1.1607	Reference	Reference	Reference	Reference

Note: For each specimen, measurement line, and sample size, 10,000 random subsets were generated without replacement from the complete set of 50 HRBW measurements. Absolute relative deviations were calculated with respect to the corresponding *n* = 50 estimate.

**Table 8:** Comparison of the complementary characterization methods used in the present study.

Method	Directly Evaluated Quantity	Spatial Information	Destructive Character	Role in the Present Study
Transverse tensile testing [52]	Tensile response, elongation, and fracture location	Global specimen-level response	Destructive	Comparison of the five welding conditions and identification of Specimens 1 and 3 as contrasting mechanical-response cases
Optical metallography [30,53,54,55,56]	Microstructural morphology of the BM, HAZ, fusion-boundary region, and FZ	Local/region-specific	Requires metallographic specimen preparation	Qualitative comparison of the microstructural morphology of Specimens 1 and 3 and metallurgical context for interpretation of the mechanical and macrohardness results
Rockwell macrohardness [46,47]	Local indentation hardness	Spatially resolved	Produces permanent local indentations; minimally destructive	Direct HRBW mapping at identical spatial positions in Specimens 1 and 3
CV(*ν*) and WHC(*m*) analysis [6,39,40]	Statistical dispersion of repeated HRBW measurements	High-density spatial profile	Derived from Rockwell measurements	Direct comparison of spatial macrohardness dispersion between Specimens 1 and 3 and evaluation of information not evident from mean HRBW alone

## Data Availability

The original contributions presented in this study are included in the article. Further inquiries can be directed to the corresponding author.

## Abbreviations

The following abbreviations are used in this manuscript:

MHC	material homogeneity criterion
WHC(*m*)	Weibull homogeneity coefficient *m*
CV(*ν*)	coefficient of variation *ν*
FZ	fusion zone
BM	base material
HAZ	heat-affected zone
HRBW	Rockwell macrohardness
NDT	non-destructive testing
